# An observational human study investigating the effect of anabolic androgenic steroid use on the transcriptome of skeletal muscle and whole blood using RNA-Seq

**DOI:** 10.1186/s12920-023-01512-z

**Published:** 2023-05-03

**Authors:** Alexander Kolliari-Turner, Giscard Lima, Guan Wang, Fernanda Rossell Malinsky, Antonia Karanikolou, Gregor Eichhorn, Kumpei Tanisawa, Jonathan Ospina-Betancurt, Blair Hamilton, Paulette Y.O. Kumi, Jonathan Shurlock, Vasileios Skiadas, Richard Twycross-Lewis, Liam Kilduff, Renan Paulo Martin, Garrett I. Ash, Cynthia Potter, Fergus M. Guppy, Jane T. Seto, Chiara Fossati, Fabio Pigozzi, Paolo Borrione, Yannis Pitsiladis

**Affiliations:** 1 School of Sport and Heath Sciences, University of Brighton Welkin House, 30 Carlisle Road, Eastbourne, BN20 7SN UK; 2 Centre for Stress and Age-Related Disease, University of Brighton, Brighton, UK; 3 Muscle Research, Murdoch Children’s Research Institute, Parkville, VIC Australia; 4 Department of Movement, Human and Health Sciences, University of Rome “Foro Italico”, Rome, Italy; 5 Centre for Regenerative Medicine and Devices, University of Brighton, Brighton, UK; 6 Environmental Extremes Laboratory, University of Brighton, Eastbourne, UK; 7 Faculty of Sport Sciences, Waseda University, Tokorozawa, Japan; 8 Faculty of Education, University of Valladolid, Soria, Spain; 9 The Gender Identity Clinic, Tavistock and Portman NHS Foundation Trust, London, UK; 10 Centre for Sports and Exercise Medicine, William Harvey Research Institute, Queen Mary University of London, London, UK; 11 Somerset NHS Foundation Trust, Taunton, UK; 12 University Hospital Southampton NHS Foundation Trust, Southampton, UK; 13 School of Engineering and Materials Science, Queen Mary University of London, London, UK; 14 St Mary’s University, Twickenham, London, UK; 15 Applied Sports, Technology, Exercise, and Medicine Research Centre (A-STEM), Faculty of Science and Engineering, Swansea University, Swansea, Wales; 16 Department of Biophysics, Federal University of Sao Paulo, Sao Paulo, Brazil; 17 McKusick-Nathans Department of Genetic Medicine, Johns Hopkins University School of Medicine, Baltimore, MD USA; 18 Veterans Affairs Connecticut Healthcare System, West Haven, CT USA; 19 Center for Medical Informatics, Yale University, New Haven, CT USA; 20 MGI, Riga, Latvia; 21 Institute for Life and Earth Sciences, School of Energy, Geoscience, Infrastructure and Society, Heriot-Watt University, Edinburgh, UK; 22 Department of Paediatrics, University of Melbourne, Parkville, VIC Australia

**Keywords:** Anabolic androgenic steroids, Doping, Skeletal muscle, Whole blood, RNA-Seq, Gene expression, Hypertrophy.

## Abstract

**Background:**

The effects of Anabolic Androgenic Steroids (AAS) are largely illustrated through Androgen Receptor induced gene transcription, yet RNA-Seq has yet to be conducted on human whole blood and skeletal muscle. Investigating the transcriptional signature of AAS in blood may aid AAS detection and in muscle further understanding of AAS induced hypertrophy.

**Methods:**

Males aged 20–42 were recruited and sampled once: sedentary controls (C), resistance trained lifters (RT) and resistance trained current AAS users (RT-AS) who ceased exposure ≤ 2 or ≥ 10 weeks prior to sampling. RT-AS were sampled twice as Returning Participants (RP) if AAS usage ceased for ≥ 18 weeks. RNA was extracted from whole blood and trapezius muscle samples. RNA libraries were sequenced twice, for validation purposes, on the DNBSEQ-G400RS with either standard or CoolMPS PE100 reagents following MGI protocols. Genes were considered differentially expressed with FDR < 0.05 and a 1.2- fold change.

**Results:**

Cross-comparison of both standard reagent whole blood (N = 55: C = 7, RT = 20, RT-AS ≤ 2 = 14, RT-AS ≥ 10 = 10, RP = 4; N = 46: C = 6, RT = 17, RT-AS ≤ 2 = 12, RT-AS ≥ 10 = 8, RP = 3) sequencing datasets, showed that no genes or gene sets/pathways were differentially expressed between time points for RP or between group comparisons of RT-AS ≤ 2 vs. C, RT, or RT-AS ≥ 10. Cross-comparison of both muscle (N = 51, C = 5, RT = 17, RT-AS ≤ 2 = 15, RT-AS ≥ 10 = 11, RP = 3) sequencing (one standard & one CoolMPS reagent) datasets, showed one gene, *CHRDL1*, which has atrophying potential, was upregulated in RP visit two. In both muscle sequencing datasets, nine differentially expressed genes, overlapped with RT-AS ≤ 2 vs. RT and RT-AS ≤ 2 vs. C, but were not differentially expressed with RT vs. C, possibly suggesting they are from acute doping alone. No genes seemed to be differentially expressed in muscle after the long-term cessation of AAS, whereas a previous study found long term proteomic changes.

**Conclusion:**

A whole blood transcriptional signature of AAS doping was not identified. However, RNA-Seq of muscle has identified numerous differentially expressed genes with known impacts on hypertrophic processes that may further our understanding on AAS induced hypertrophy. Differences in training regimens in participant groupings may have influenced results. Future studies should focus on longitudinal sampling pre, during and post-AAS exposure to better control for confounding variables.

**Supplementary Information:**

The online version contains supplementary material available at 10.1186/s12920-023-01512-z.

## Background

Anabolic Androgenic Steroids (AAS) increase fat free mass, muscle size and strength in men [1, 2] with the majority of hypertrophic effects mediated through the Androgen Receptor (AR) [3] which induces a genomic mode of action by modulating transcription [4]. AAS are known to have a rapid non-genomic AR-independent mode of action that influences cellular behaviour, by increasing intracellular Ca^2+^ concentration through the activation of a G-protein-linked receptor at the plasma membrane [5], however, in human skeletal muscle, the physiological significance of the rapid non-genomic action of AAS for hypertrophy remains unclear [6, 7].

Due to these ergogenic effects, AAS are amongst the most widely detected doping substances in strength and power sports [8, 9] and their detection accounts for 67% of all the medal-winning results impacted by Anti-Doping Rule Violations at the Summer Olympic Games 1968–2012 [10]. The next generation “omic” approach to detect doping is based on the premise that doping methods will cause profound and, therefore, detectable changes in the ways genes are expressed and thereby generate a unique “omic signature” of exposure to a specific doping practice [11]. This “omic signature” is thought to be difficult to mask and deemed to have the potential to significantly improve the reliability and extend the window of detection of doping tests [11]. For example, both high [12] and low dose [13] recombinant human erythropoietin (rHuEPO) administration studies have shown a whole blood transcriptional signature that has a more prolonged detection window for rHuEPO doping compared to traditional methods [13] that is not confounded by exercise [13] or altitude exposure [13, 14], with this transcriptional signature shown across two microarray platforms (Affymetrix and Illumina) and two RNA-Seq platforms (Illumina and MGI) [15]. Whole blood stored in K_2_EDTA tubes, which lack RNA preservative, still yielded RNA of sufficiently high quantity, purity, and integrity for transcriptomic analysis with no impact on genes previously identified in rHuEPO administration studies, potentially indicating that transcriptomic analysis could be integrated into the current anti-doping system, by utilising remaining/excess blood from routine testing [16].

Since the AR is expressed in whole blood [17], RNA-Seq could be used to screen for transcriptomic biomarkers that could be beneficial additions to the steroidal module of the Athlete Biological Passport (ABP) [18] to enhance doping detection. A low-dose testosterone administration study has shown that circulating micro-RNA 122 (miR-122) could act as a potential transcriptomic biomarker, with miR-122 levels significantly higher 24-hours after testosterone administration compared to both baseline and a control group [19]. This detection window is longer than that of individual monitoring of typical urinary metabolites (2–12 h) for oral testosterone administration [19]. RNA-Seq of liver samples from AAS-treated and untreated calves and boars has also successfully identified biomarker candidates that could distinguish AAS treatment [20], further indicating that AAS transcriptomic signatures could aid doping detection.

This study aims to build on previous findings of potential transcriptomic biomarkers of AAS by using RNA-Seq on RNA extracted from whole blood from both current and past AAS users, in addition to control groups of resistance trained and un-trained males. RNA-Seq of muscle samples will also be performed to further our knowledge of the genomic mode of action of AAS in hypertrophic pathways and complement previous research on this cohort of participants on muscle fibre parameters related to hypertrophy and muscle memory [21]. To the authors knowledge RNA-Seq has not been conducted on human whole blood or skeletal muscle samples from current or past AAS users which is one of the contributions of this study to the field of AAS research.

## Methods

### Eligibility criteria and group classification

Participants were male, aged 20–42 and were initially recruited to fall within one of four groups, according to their self-reported resistance training and AAS usage history (Table 1), as reported in a previous publication [21] of body composition and immunohistochemistry data on this cohort. If participants within RT-AS self-reported to having refrained from AAS usage for at least 18 weeks after their first visit, they were invited back for a second visit attempting to give the closest possible comparison to a situation in which AAS were completely removed. All participants were interviewed regarding their AAS usage prior to sample collection on their first visit. Returning Participants (RP) were interviewed prior to their second visit to discuss their training, nutrition, and Post Cycle Therapy (PCT) protocol [22, 23] which is commonly used in an attempt to re-stimulate endogenous testosterone production and manage the removal of AAS. Any participant that declared using Testosterone Replacement Therapy (TRT) was not re-sampled as this could surpass current TRT guidelines (i.e., > 100 mg/week) [24, 25] and thereby confound the data collected post initial AAS usage.

**Table 1:** Group allocation criteria used during initial recruitment of participants

Group	Criteria
**C**	1) A control group comprised of non-resistance trained healthy males.
**RT**	1) History of resistance training ≥ 8 h a week 2) Self-reported as never using any PEDs.
**RT-AS**	1) History of resistance training ≥ 8 h a week 2) Self-reported to using exogenous AAS (i.e., synthetic AAS), supraphysiological dosages of injectable testosterone (> 100 mg/week) or a closely related AAS compound (i.e., AAS pro-hormones or SARMs) < 52 weeks prior to their first sampling date.
**PREV**	1) History of resistance training ≥ 8 h a week 2) Self-reported to ceasing usage of exogenous AAS (i.e., synthetic AAS), supraphysiological dosages of injectable testosterone (> 100 mg/week) or a closely related AAS compound (i.e., AAS pro-hormones or SARMs) ≥ 52 weeks prior to their sampling date and not exceeded any clinical recommendations for TRT(24, 25) during this period of absence.

AAS: Anabolic androgenic steroids; PED: Performance Enhancing Drug; SARMs: Selective Androgen Receptor Modulators; TRT: Testosterone Replacement Therapy

The full self-reported lifetime history of AAS cycles from participants within group RT-AS and PREV have been published elsewhere [21].

Participants in RT-AS and PREV were subsequently subdivided to those where last self-declared AAS exposure was less than or equal to two weeks prior to sample collection (RT-AS ≤ 2) and those where self-declared last AAS exposure was 10 or more weeks prior to sample collection (RT-AS ≥ 10). This is because the process of a steroid receptor translocating from the cytoplasm to the nucleus typically takes at least 30–60 min [6] and thus time since last exposure is a pertinent variable to classify participants when investigating potential differences in gene expression. The last total weekly dose of AAS used was noted down at interview and if the last reported exposure of AAS occurred over a 7-day period (e.g., an injection every 10th day) the daily average over this period would be taken and multiplied by 7 and rounded to the nearest 10. If the participant reported a dosage range of AAS used per week (e.g., 350-420 mg) the average of the range would be used. Group data are presented as mean ± standard deviation.

### Blood Collection & muscle biopsy

Participants were instructed to not resistance train 48-hours prior to the biopsy and they ate normally before visiting the laboratory due to the medical advice that participants could feel dizziness during the biopsy, in which they were seated upright on a chair with no backing. 3 mL of whole blood was collected into a Tempus™ Blood RNA Tube (Life Technologies, Carlsbad, CA, USA) from an antecubital vein utilising a closed vacuette system 2–6 h prior to the muscle biopsy, with participants iteratively sampled, in the order of consenting, at regular intervals throughout a working day. Immediately after collection the tube was manually vigorously shaken for 10 s, left at room temperature for 3 h and then stored at − 80 °C.

All muscle biopsies were performed by an experienced Consultant Musculoskeletal Radiologist. The upper part of the trapezius muscle (descending I) was the chosen site of the muscle biopsy, as detailed in previous research [26–28]. The ultrasound guided biopsy technique utilising a BARD® Magnum® Disposable Core Biopsy Needle has been detailed in a previous publication on this cohort of participants [21]. The first sample of four collected samples, that was placed inside Qiagen® RNAlater RNA Stabilization Reagent (76106, Qiagen®, Hilden, North Rhine-Westphalia, Germany) was used for RNA extraction in this present study.

### RNA extraction and purification

Blood and muscle samples were randomly sorted prior to RNA extraction and library preparation. Total RNA was extracted from whole blood using the Tempus™ Spin RNA Isolation Kit according to manufacturer instructions (Life Technologies, Carlsbad, CA, USA). Total RNA > 200 nt was extracted from muscle samples using the Qiagen® RNeasy Fibrous Tissue Mini Kit with TissueRuptor II Disposable Probes. Muscle total RNA samples were then digested using DNase I (New England Biolabs, Ipswich, MA, USA) and purified using RNAClean XP beads (Beckman Coulter, Indianapolis, IN, USA). After extraction, all RNA samples were stored at − 80 °C until further analysis.

### RNA Quality Assessment

RNA quantity and quality were assessed using a Nanodrop® ND-2000 Spectrophotometer (Thermo Fisher Scientific, Waltham, MA, United States). RIN value was assessed using an Agilent® 2100 Bioanalyzer with an Agilent® RNA 6000 Nano Kit (Agilent Technologies, Santa Clara, CA, United States).

### RNA Library Preparation and RNA-Seq

rRNA was depleted from 200 ng of total whole blood RNA or purified total muscle RNA with RIN ≥ 7 using an MGIEasy rRNA Depletion Kit. dsDNA libraries (with conditions for a 250-bp Insert Size) were created from the rRNA-depleted eluate using an MGIEasy RNA Directional Library Prep Set. dsDNA library quantity was assessed using a Thermo Fisher Scientific Qubit® dsDNA High Sensitivity Assay Kit and a Qubit® Fluorometer (Thermo Fisher Scientific, Waltham, MA, United States). The quality of the fragment size distribution of the dsDNA library was assessed by visual inspection of electropherograms created using an Agilent® DNA 1000 Kit on an Agilent® 2100 Bioanalyzer. Only dsDNA libraries with satisfactory fragment size distributions were carried forward onto the next steps, and dsDNA libraries were recreated for any samples with aberrant electropherograms or low concentrations. dsDNA libraries were circularized and converted into ssDNA libraries using an MGIEasy Circularization Kit. ssDNA library concentration was assessed using a Thermo Fisher Scientific Qubit® ssDNA Assay Kit and a Qubit® Fluorometer. DNA nanoballs (DNBs) were prepared from ssDNA library pools, with a 40-fmol ssDNA library for each reaction, using an MGI DNBSEQ-G400RS High-throughput Sequencing Set for blood samples and for muscle samples either this kit or a CoolMPS High-throughput Sequencing Set. DNB concentration was assessed using a Qubit® ssDNA Assay Kit (Thermo Fisher Scientific) and a Qubit® Fluorometer (Thermo Fisher Scientific). DNB preparations > 8 ng/µL were loaded onto flow cells using an MGIDL-200 H Portable DNB Loader, with muscle and whole blood samples distributed over two flow cells each, with six to eight samples in each lane based on their order of RNA extraction and library preparation. The flow cells were placed on an MGI DNBSEQ-G400 sequencer and subjected to PE100 sequencing with standard chemistry reagents for blood samples and either standard chemistry reagents or CoolMPS chemistry reagents for muscle samples. Two flow cells of whole blood samples were sequenced at the UoB, School of Sport and Health Sciences campus in Eastbourne. For validation purposes these same libraries were re-sequenced in MGI’s research hub in Latvia. Two flow cells of muscle samples were sequenced with standard chemistry reagents in MGI’s research hub in Latvia and for validation purposes these same libraries were re-sequenced with CoolMPS chemistry reagents in MGI’s research hub in Latvia.

### Sequencing data Quality Control

Raw sequences were examined by FastQC [29] version 0.11.9 for basic quality checks (e.g., per base sequence quality, per base N content, duplicate sequences and adapter content). RSeQC [30] version 4.0.0 and the function read_duplication.py was also utilised for further quality checks. FastQ Screen [31] version 0.15.0 was used for detecting sample swaps and/or sample contamination utilising Bowtie 2 [32] version 2.4.2 for alignment to reference genome assemblies with pre-built Bowtie 2 genome indices [33] for Human (GRCh38 no-alt analysis set), Mouse (GRCm39) and Rat (Rnor6.0). MultiQC [34] was used to summarise FastQC, FastQ Screen and compatible RSeQC analysis reports. FastQC per base sequence quality scores, interactive MultiQC reports for FastQ Screen and RSeQC are available on OSF [35].

### Read mapping, transcript quantification and differential expression analysis

HISAT2 [36] version 2.2.1 was used for alignment of reads to the reference genome assembly GRCh38.p5 using the Ensembl 84 annotation as the publicly available grch38_tran pre-built HISAT2 index [37, 38] was utilised. For HISAT2 alignment ‘--dta’ was utilised and ‘--rna-strandness RF’ was stated as RSeQC infer_experiment.py showed a directional, first strand library. Galaxy [39] was used to convert the Homo_sapiens.GRCh38.84.gtf.gz file to the BED12 file format for RSeQC infer_experiment.py. RSeQC read_distribution.py was used for read distribution analysis also utilized this BED12 file and SAM files generated from HISAT2. RSeQC split_bam.py was used to estimate how many reads originated from rRNA utilising the publicly available hg38_rRNA.bed file [40] from RSeQC. Salmon [41] version 1.7.0 was used for transcript quantification in mapping-based mode utilising the publicly available hg38 full decoy-aware salmon index [42] with --validateMappings, --seqBias and --gcBias flags switched on. Using Bioconductor version 3.14 and R [43] version 4.1.2 in Rstudio [44] version 2022.2.0.443 the package “tximport” [45] was used for summarising transcript-level estimates to gene names based on the Ensembl release 105 [46] annotation and transcript IDs with undefined gene names were removed. Data was normalised by the calcNormFactors() function in edgeR [47], explored with MDS and PCA Plots and if deemed appropriate, based on aberrant positioning, outlying samples were removed and data re-normalised. Data was then filtered by group for group comparisons or by visit for paired sample comparisons in returning participants. For group comparisons only first visit data from RP1-5 was utilised. For group comparisons the minimum number of counts per sample matched the smallest group size and for paired sample comparisons for returning participants, the minimum number of counts per sample matched the total number of samples in the dataset. Data was then re-normalised, experimental designs were then modelled (i.e., group comparisons or paired sample comparisons), dispersion estimates were calculated and then the quasi-likelihood approach was used to fit generalised linear models to the data. Group contrasts or paired sample contrasts were made, and DGE testing was conducted. The function topTags() was used to select the most differentially expressed genes using a false discovery rate (FDR) < 0.05 and a fold change of 1.2.

### Gene Set Enrichment Analysis and hierarchical clustering

Gene Set Enrichment Analysis (GSEA) was conducted in R using the Bioconductor package GSEABase [48] version 1.56.0 and fry [49] by examining the Molecular Signatures Database [50, 51] (MSigDB) v7.5.1 Hallmark (containing 50 gene sets) [52], Gene Ontology [53, 54] (C5; BP: subset of GO biological processes containing 7,658 gene sets & MF: subset of GO molecular functions containing 1,738 gene sets), KEGG [55] pathway (186 gene sets) and Reactome [56] pathway (1615 gene sets) collections. A gene set/pathway was noted as differentially expressed if FDR < 0.05. For each comparison, lists of differentially expressed genes and gene sets/pathways were exported into InteractiVenn [57] to identify overlaps between different sequencing locations (blood samples, UoB & MGI) or sequencing chemistries (muscle samples, standard & CoolMPS).

Gene and sample clustering was performed within pheatmap [58]. Firstly, normalised counts per million were log transformed for the top 30 most significantly differentially expressed genes by FDR for the group comparison noted. This matrix was inputted into pheatmap [58]; rows and columns were clustered with a complete clustering method, Pearson correlation was used as the distance measure and scale = “row” was applied.

## Results

### Participant sampling and AAS usage

**Fig. 1:**
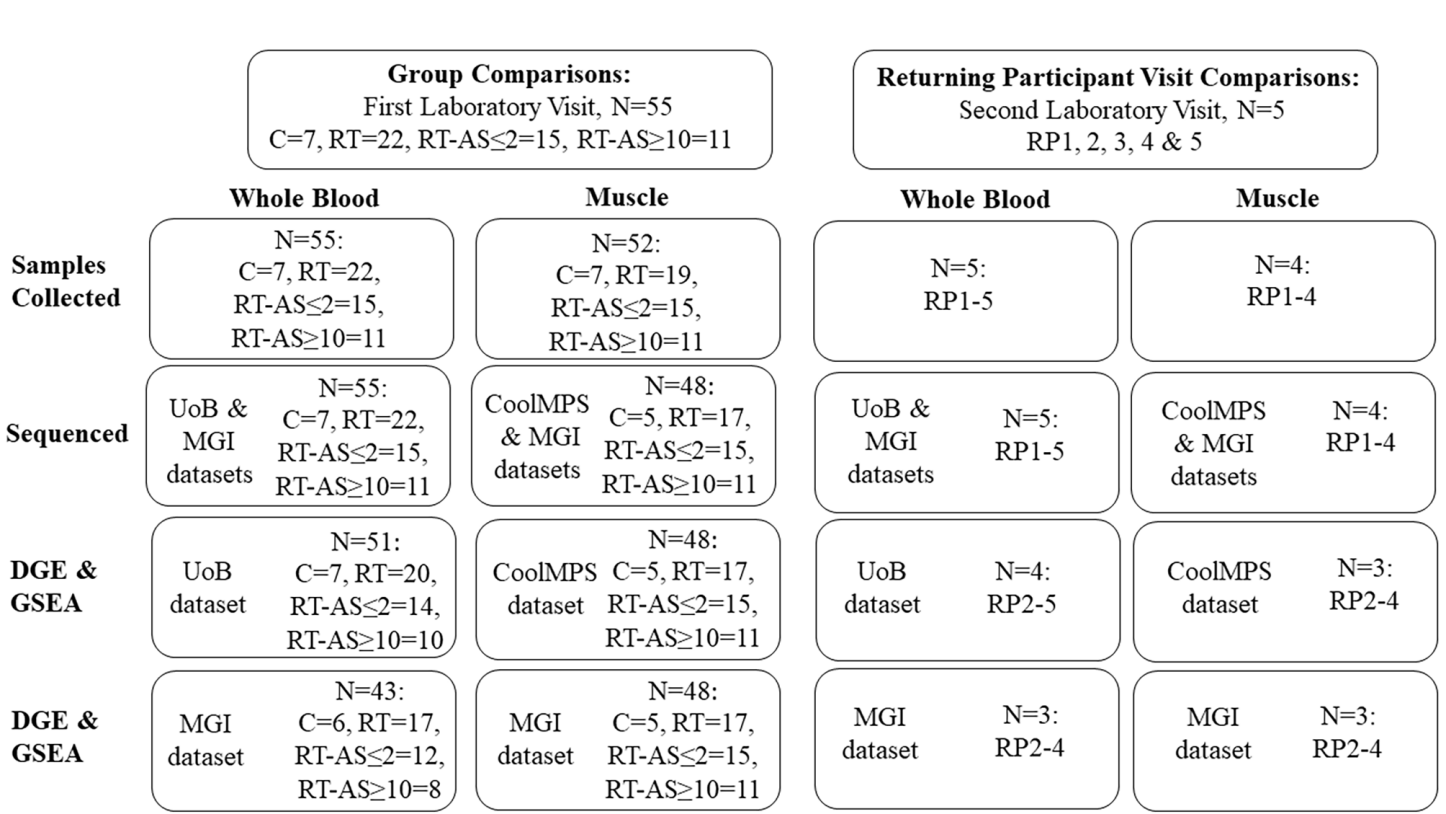
Participant sampling and sample inclusion for group and returning participant comparisons. Returning Participant Visit 1 is included within their corresponding RT-AS cohort. C = Control; RT = Resistance Trained; RT-AS ≤ 2 = Resistance Trained participant who self-declared AAS exposure ceased ≤ 2 weeks before sampling; RT-AS ≥ 10 = Resistance Trained participant who self-declared AAS exposure ceased ≥ 10 weeks before sampling; RP = Returning Participant; DGE: differential gene expression; GSEA: Gene Set Enrichment Analysis; UoB: indicates samples sequenced at the University of Brighton utilising standard chemistry reagents; MGI: indicates samples sequenced at MGI, Latvia, utilising standard chemistry reagents; CoolMPS: indicates samples sequenced at MGI, Latvia, utilising CoolMPS chemistry reagents

Fifty-five participants were sampled on first laboratory visit (Fig. 1). Of those participants within RT-AS ≤ 2 (n = 15), ten declared using AAS the week of sampling, four declared ceasing usage 1-week prior and one 2-weeks prior with an average last recorded weekly AAS dosage of 489 ± 319 mg and range of 175 mg – 1,300 mg. Of those participants within RT-AS ≥ 10 (n = 11), the number of weeks since last self-declared AAS exposure ranged from 10 to 347 weeks, with one participant declaring ceasing usage 10 weeks ago and the remaining ten declaring ceasing usage ≥ 34 weeks ago with an average last recorded weekly AAS dosage of 424 ± 226 mg and range of 175 mg – 700 mg.

Five participants within RT-AS returned (RP1-5) for a second laboratory visit post exposure (Fig. 1). Four of these participants (RP2-5) finished exposure ≤ 2 weeks prior to their first visit and had 28, 28, 19 and 22 weeks, respectively between visits. The last recorded weekly dose of AAS used was 505 ± 236 mg for 7.8 ± 1.8 weeks for RP2-5. RP1 used 700 mg of AAS for 10 weeks, his first visit was 34 weeks after exposure, and his second visit 28 weeks later. Due to the differing timescale of cessation relative to sampling for RP1 compared to RP2-5, RP1 was excluded from returning participant comparisons. Three participants (RP1, RP3 and RP4) self-reported only using PCT compounds and no other PEDs between visits while two participants (RP2 and RP5), self-reported using either Ibutamoren or Clenbuterol between visits [21].

### RNA quantity, purity, and Integrity

For all whole blood samples (n = 60, Fig. 1) extracted total RNA was of sufficient concentration (103.1 ± 33.70 ng/µL), purity (A_260_/A_280_ 2.09 ± 0.02; A_260_/A_230_ 2.35 ± 0.1) and integrity (RIN 8.88 ± 0.57) for the thresholds stated in the used RNA library preparation protocols and thereby were subjected to RNA-Seq. After DNase I digestion four muscle samples (C = 2, RT = 2) did not have sufficient RNA yield for RNA library preparation. The remaining muscle samples (n = 52, Fig. 1) had RNA that was of sufficient concentration (46.16 ± 19.59 ng/µL), purity (A_260_/A_280_ 2.05 ± 0.05; A_260_/A_230_ 1.81 ± 0.16) and integrity (RIN 8.34 ± 0.5) for the thresholds stated in the used RNA library preparation protocols and thereby were subjected to RNA-Seq.

### RNA-Seq Quality Control

FastQC showed that Mean Sequence Quality Scores were high (> 30) for all samples (data available on OSF [35]). Fast Q Screen showed that sample swaps to those containing other species had not occurred in any sequencing dataset (data available on OSF [35]). Six blood samples (P04, P13, P15, P36, P41, P43), in both MGI and UoB sequencing datasets (data available on OSF [35]), showed abnormally high sequence duplication levels with overrepresented sequences matching known DNA oligos [59] used for rRNA depletion. This is caused by pipetting errors in the initial steps of library preparation of these samples in which an insufficient quantity of DNase enzyme was added that would digest added DNA oligos used for rRNA depletion. Four of these samples (P04, P15, P41, P43), that had the highest levels of reads mapping to other intergenic regions with RSeQC, were further deemed as outliers based on MDS & PCA Plots (Additional File 1 Fig. 1a and b) of all blood samples (n = 55) sequenced at UoB used in group comparisons and were subsequently removed from further downstream analysis (Fig. 1). MDS & PCA Plots (Additional File 1 Fig. 2a and b) of all blood samples (n = 55) sequenced at MGI used in group comparisons showed, compared to the UoB dataset, a cluster of 8 samples sequenced on Flow Cell Lane B1 (P13, P17, P20, P26, P32, P44, P49, RP5 Visit 1) and from this finding and the notion lane position was a randomised order were removed as outliers. The subsequent MDS & PCA Plots (Additional File 1 Fig. 3a and b) of remaining blood samples (n = 47) sequenced at MGI used in group comparisons was similar to the UoB MDS & PCA Plots (Additional File 1 Fig. 1a and b) and for consistency between UoB and MGI datasets the four samples (P04, P15, P41, P43) with aberrant library preparation were also removed from further downstream analysis from the MGI dataset as outliers (Fig. 1). MDS & PCA Plots of blood samples (n = 8) of RP2-5 1^st^ and 2^nd^ visits from the UoB and MGI datasets (Additional File 1 Figs. 4a and b and 5a and b) showed that RP5 Visit 1, like in the MGI group comparison data set, was an outlier and so due to paired sample analysis RP5 was removed from further downstream analysis in the MGI returning participant dataset (Fig. 1). The subsequent MDS & PCA Plots (Additional File 1 Fig. 6a & b) of the MGI dataset of RP2-4 (n = 6) 1^st^ and 2^nd^ visits were similar to the UoB dataset of all (n = 8) RP samples (Additional File 1 Fig. 4a and b).

No muscle samples were excluded from downstream analysis (Fig. 1). No abnormally high levels of sequence duplication levels were observed, and no overrepresented sequences matched DNA oligos used for rRNA depletion. MDS & PCA Plots of the standard and CoolMPS chemistry sequencing datasets, used in group comparisons (Additional File 1 Figs. 7a and b and 8a and b) and RP Visit comparisons (Additional File 1 Figs. 9a and b and 10a and b), showed no obvious outliers and were similar. The RSeQC function split_bam.py showed that all blood and muscle samples had zero reads originating from rRNA showing that rRNA depletion was successful.

### Read mapping, read distribution, and transcript quantification

Genome mapping using HISAT2 [36] respectively showed average overall alignment rates of 96.6 ± 1.2% and 95.4 ± 1.9% for whole blood samples sequenced at UoB (n = 55) and MGI (n = 46) and 95.3 ± 1.7% and 98.4 ± 0.4% for muscle (n = 51) samples sequenced with standard and CoolMPS reagents (Additional File 2 Tables 1, 2, 3 and 4) that were used in downstream DGE/GSEA analyses.

RSeQC [30] showed a higher proportion of reads in whole blood samples sequenced at UoB (n = 55, 26.4 ± 4.5%) and MGI (n = 46, 26.2 ± 4.7%) used in downstream DGE/GSEA analyses mapped to introns compared to muscle (n = 51) samples used in downstream DGE/GSEA analyses sequenced with standard and CoolMPS reagents (17.1 ± 2.7% and 17.2 ± 2.6%) (Additional File 2 Tables 5, 6, 7 and 8). These differences are likely related to differences in the RNA extraction protocols used (extracting total RNA in whole blood vs. extracting total RNA > 200 nucleotides in length in muscle).

For whole blood samples sequenced at UoB (n = 55) and MGI (n = 46) used in downstream DGE/GSEA analyses the average number of processed reads (59.5 ± 15.4 million and 59.9 ± 11.5 million) and mapped reads (55.4 ± 6.9% and 55.6 ± 6.4%) used by Salmon was lower than in muscle samples (n = 51) sequenced with standard (69.3 ± 12.2 million, 70.2 ± 3.8%) and CoolMPS (68.2 ± 11.6 million, 70.5 ± 3.7%) reagents (Additional File 2 Tables 9, 10, 11 and 12).

The number of genes available for DGE analysis, across the four analysed datasets, was 14,353–16,687 (Additional File 2 Table 13a - d) following the stated count filtering criteria after Salmon transcript-level estimates were summarised to genes.

The biological coefficient of variation (BCV) for whole blood samples sequenced at UoB and MGI is greater (0.26) than the 0.01 threshold stated in the edgeR user manual [60] as an acceptable amount of variation for technical replicates and thereby they have not been merged as one dataset and instead have been used to cross-validate each other. As standard reagent and CoolMPS reagent sequencing chemistries differ [61] these two muscle datasets cannot be merged as technical replicates and so have been used to cross-validate each other.

### Differential Gene Expression analysis – returning participants

For returning participant visit comparisons no differentially expressed genes were identified in the UoB (n = 8) sequencing dataset, although 11 differentially expressed genes were identified in the MGI (n = 6) sequencing dataset and so none overlapped between sequencing datasets (Additional File 2 Table 14a & 14b). Returning participants clustered by participant in MDS & PCA Plots (Additional File 1 Figs. 4a and b and 6a and b).

Of the six genes identified as differentially expressed, across both muscle sequencing datasets (Additional File 2 Table 14c & 14d), from returning participants first and second visits (n = 6), only one of these genes was differentially expressed in both datasets (Table 2) with *CHRDL1* being upregulated in Visit 2. Returning participants clustered by participant in MDS & PCA Plots (Additional File 1 Figs. 9a and b and 10a and b).

**Table 2:** Number of differentially expressed genes that overlap for muscle samples sequenced with standard and CoolMPS reagents at MGI subjected to DGE analysis across different group comparisons

Comparison							
RP2-4 Visit 2 (n = 3) vs. Visit 1 (n = 3)	RT (n = 17) vs. C (n = 5)	RT-AS ≤ 2 (n = 15) vs. C (n = 5)	RT-AS ≥ 10 (n = 11) vs. C (n = 5)	RT-AS ≤ 2 (n = 15) vs. RT (n = 17)	RT-AS ≥ 10 (n = 11) vs. RT (n = 17)	RT-AS ≤ 2 (n = 15) vs. RT-AS ≥ 10 (n = 11)	Up/Down regulation
1	2	18	2	68	0	11	Up
0	1	20	0	37	0	6	Down

Genes with a false discovery rate (FDR) < 0.05 and fold change of 1.2 were reported as differentially expressed. DGE: differential gene expression; UoB: University of Brighton; RP: Returning Participant; C: non-resistance trained control group; RT: Resistance Trained control group RT-AS ≤ 2: Resistance Trained participant who self-declared AAS exposure ceased ≤ 2 weeks before sampling; RT-AS ≥ 10: Resistance Trained participant who self-declared AAS exposure ceased ≥ 10 weeks before sampling

### Differential Gene Expression analysis – group comparisons

Both blood sequencing datasets subjected to DGE analysis (Fig. 1) did not show clear group clustering in MDS & PCA Plots (Additional File 1 Fig. 11a,b & 12a,b). Cross comparison of DGE analysis results of both blood sequencing datasets (Additional File 2 Table 14a & 14b) only identified two genes as differentially expressed in both datasets, with *MTND1P23* downregulated when RT was compared to C and *IGLV3-10* upregulated when RT-AS ≥ 10 was compared to RT.

**Fig. 2:**
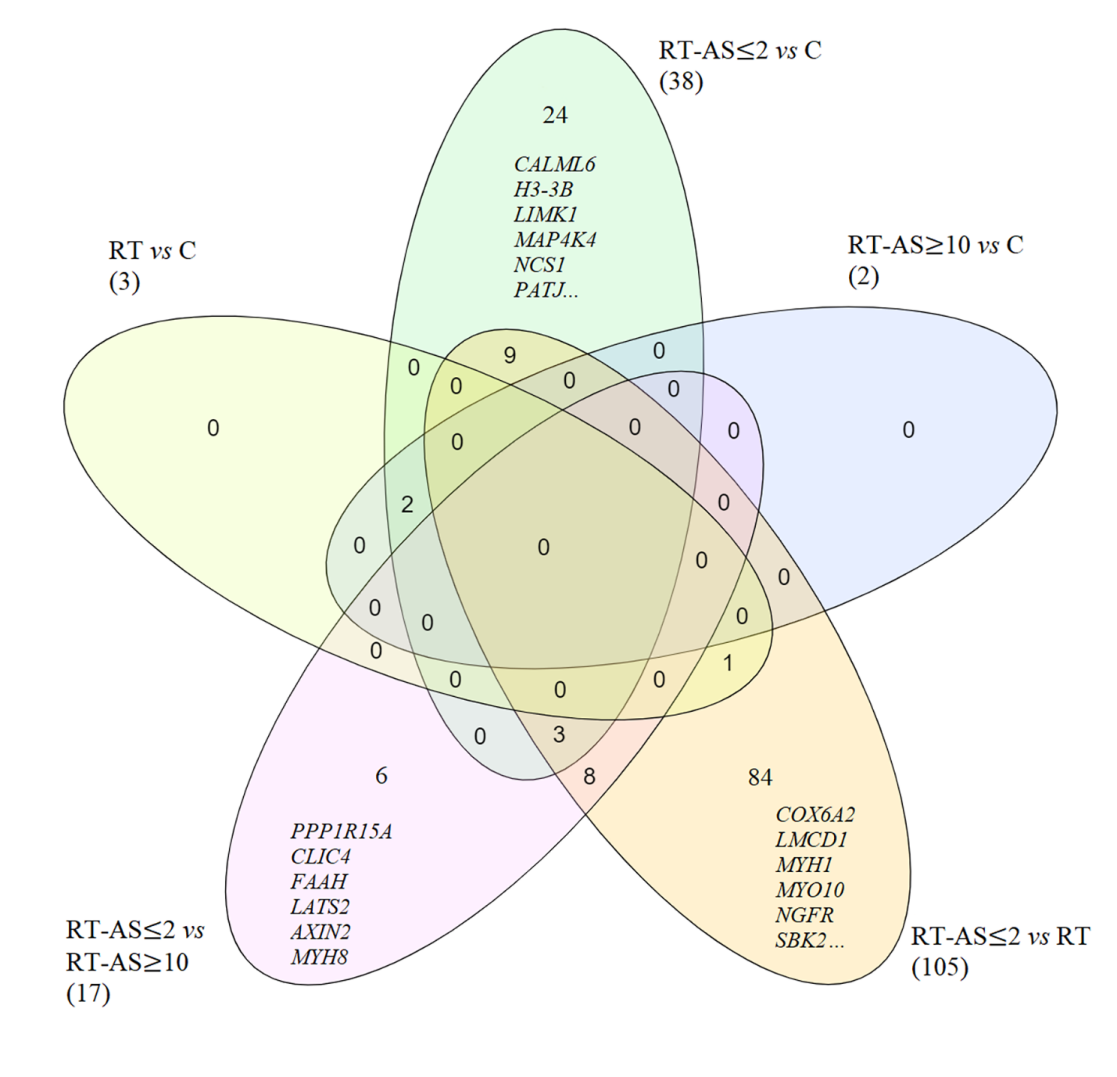
A Venn Diagram of differentially expressed genes that overlapped between the standard and CoolMPS sequencing datasets of the muscle samples with different group comparisons. Numbers in brackets indicate the total number of differentially expressed genes for that comparison. C: non-resistance trained control group (n = 5); RT: Resistance Trained control group (n = 17); RT-AS ≤ 2: Resistance Trained participant who self-declared AAS exposure ceased ≤ 2 weeks before sampling (n = 15); RT-AS ≥ 10: Resistance Trained participant who self-declared AAS exposure ceased ≥ 10 weeks before sampling (n = 11)

Both muscle sequencing datasets subjected to DGE analysis (Fig. 1) did not show clear group clustering in MDS & PCA Plots (Additional File 1 Figs. 7a and b and 8a and b). When both muscle datasets (Additional File 2 Table 14c & 14d) were cross compared, for validation purposes, each group comparison had differentially expressed genes, except for when RT-AS ≥ 10 was compared to RT when no differences were present (Table 2). The greatest number of differentially expressed genes occurred when RT-AS ≤ 2 was compared to Group RT in which 68 genes were upregulated and 37 downregulated (Table 2). Figure 2 shows, for genes that overlap across both muscle sequencing datasets, a Venn Diagram of all five group comparisons that had differentially expressed genes.

Lists of all genes and associated log Fold Change and FDR values for all returning participant and group comparisons subjected to DGE analysis are available on OSF [35]. For muscle sequencing datasets a list of all overlapping significantly differentially expressed genes (FDR < 0.05 and a 1.2- fold change) for all group comparisons is also available (Additional File 3).

### Gene Set Enrichment Analysis – returning participants

Both blood sequencing datasets for returning participants (Fig. 1), did not show any differences in the gene sets or pathways tested between visits.

Cross comparison of the two muscle sequencing datasets, showed for returning participants (n = 3, RP2-4) no differences between visits in the gene sets or pathways tested (Table 3). However, each individual dataset did identify a low number of differences, with the standard chemistry dataset having one GO BP gene set differentially expressed and the CoolMPS dataset having two Reactome pathways differentially expressed (Additional File 2 Table 15a and 15b).

**Table 3:** Number of overlapping differentially expressed gene sets or pathways for muscle samples sequenced with standard reagents and CoolMPS reagents at MGI subjected to GSEA analysis across different group comparisons

Comparison							
RP2-4 Visit 2 (n = 3) vs. Visit 1 (n = 3)	RT (n = 17) vs. C (n = 5)	RT-AS ≤ 2 (n = 15) vs. C (n = 5)	RT-AS ≥ 10 (n = 11) vs. C (n = 5)	RT-AS ≤ 2 (n = 15) vs. RT (n = 17)	RT-AS ≥ 10 (n = 11) vs. RT (n = 17)	RT-AS ≤ 2 (n = 15) vs. RT-AS ≥ 10 (n = 11)	Gene Set or Pathway
0	0	0	0	0	0	0	Hallmark
0	0	1	0	12	2	0	KEGG
0	0	0	0	74	0	1	Reactome
0	0	14	0	176	0	2	GO BP
0	0	6	0	120	0	8	GO MF

Gene sets or pathways with a false discovery rate (FDR) < 0.05 were reported as differentially expressed. GSEA: Gene Set Enrichment Analysis; UoB: University of Brighton; RP: Returning Participant; C: non-resistance trained control group; RT: Resistance Trained control group; RT-AS ≤ 2: Resistance Trained participant who self-declared AAS exposure ceased ≤ 2 weeks before sampling; RT-AS ≥ 10: Resistance Trained participant who self-declared AAS exposure ceased ≥ 10 weeks before sampling; GO BP: subset of GO biological processes; GO MF: subset of GO molecular functions

### Hierarchical clustering

**Fig. 3:**
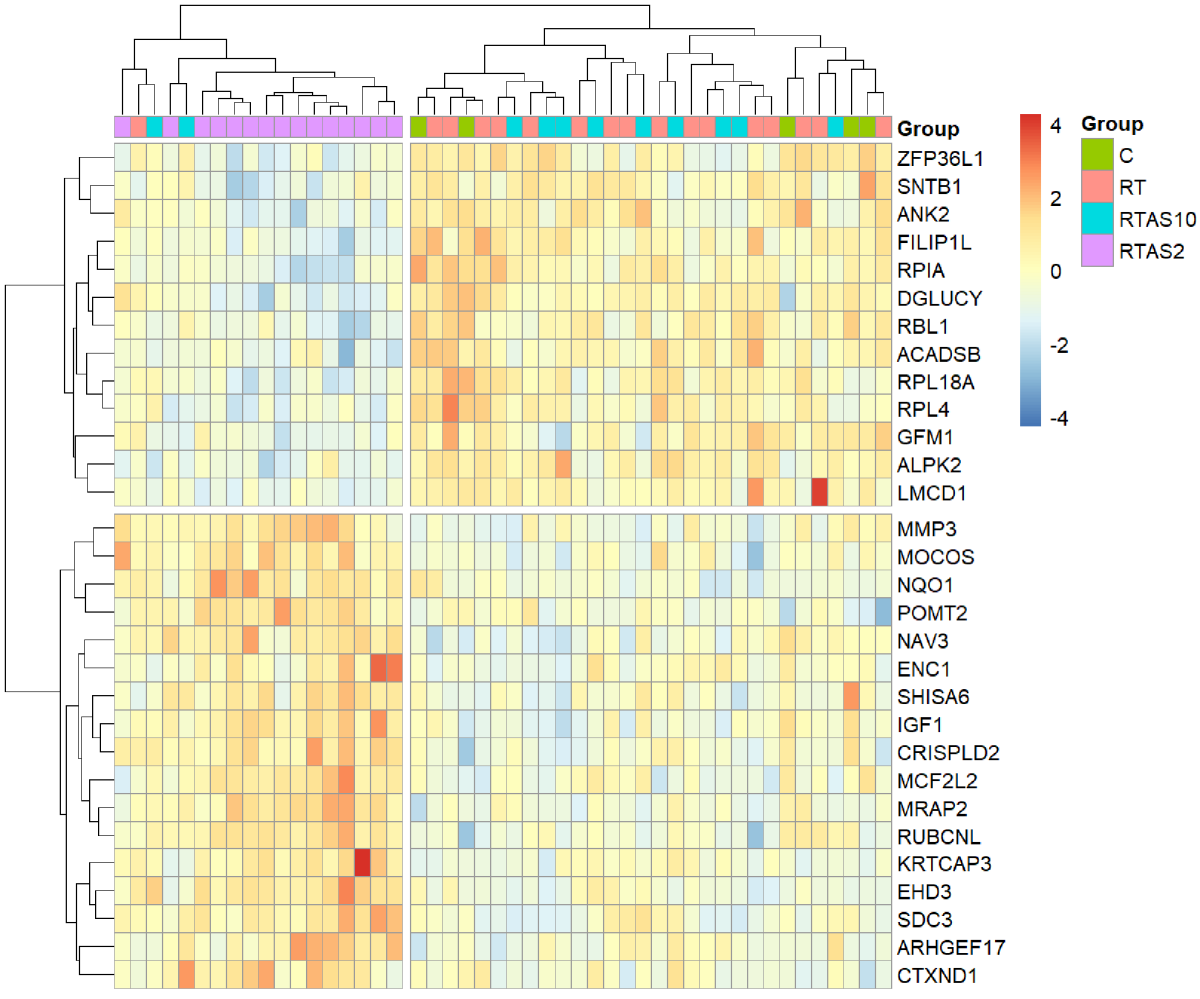
A heatmap of the top 30 most differentially expressed genes by FDR, with a minimum 1.2-fold change, from the group comparison RT-AS ≤ 2 vs. RT for all muscle samples subjected to DGE analysis sequenced with standard chemistry reagents at MGI, Latvia. Gene and sample clustering was performed within pheatmap [58]. C: non-resistance trained control group (n = 5); RT: Resistance Trained control group (n = 17); RT-AS ≤ 2: Resistance Trained participant who self-declared AAS exposure ceased ≤ 2 weeks before sampling (n = 15); RT-AS ≥ 10: Resistance Trained participant who self-declared AAS exposure ceased ≥ 10 weeks before sampling (n = 11); DGE: differential gene expression

**Fig. 4:**
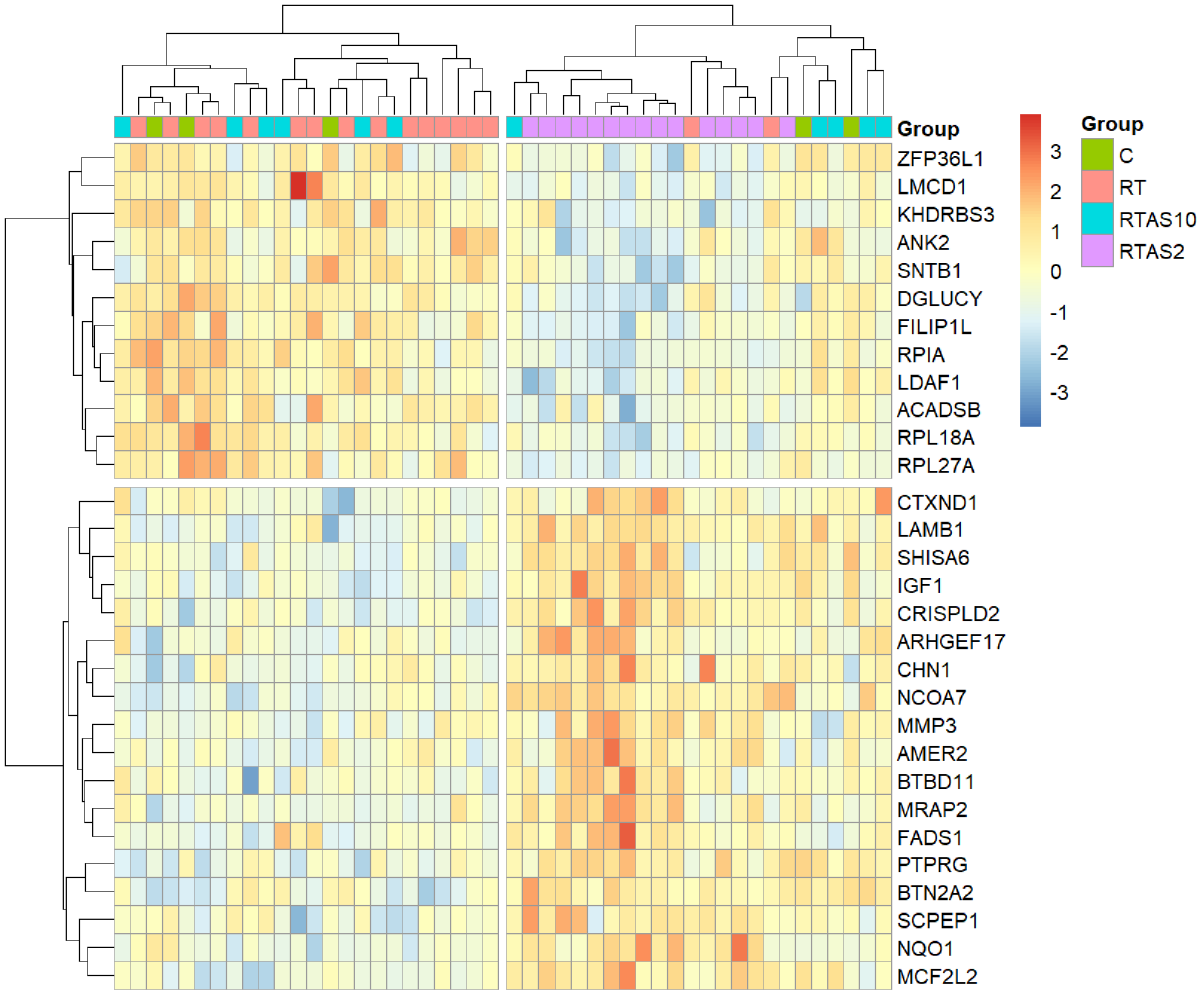
A heatmap of the top 30 most differentially expressed genes by FDR, with a minimum 1.2-fold change, from the group comparison RT-AS ≤ 2 vs. RT for all muscle samples subjected to DGE analysis sequenced with CoolMPS reagents at MGI, Latvia. Gene and sample clustering was performed within pheatmap [58]. C: non-resistance trained control group (n = 5); RT: Resistance Trained control group (n = 17); RT-AS ≤ 2: Resistance Trained participant who self-declared AAS exposure ceased ≤ 2 weeks before sampling (n = 15); RT-AS ≥ 10: Resistance Trained participant who self-declared AAS exposure ceased ≥ 10 weeks before sampling (n = 11); DGE: differential gene expression

Respectively, hierarchical clustering of muscle samples and the top 30 most significantly differentially expressed genes by FDR, with a minimum 1.2-fold change, for the group comparison RT-AS ≤ 2 (n = 15) vs. RT (n = 17) for standard (Fig. 3) and CoolMPS (Fig. 4) datasets showed that samples within RT-AS ≤ 2 tended to cluster together with a subset of genes being down and upregulated.

### Gene Set Enrichment Analysis – group comparisons

For blood samples sequenced at UoB subjected to GSEA (Fig. 1), only two GO BP gene sets showed differences when RT-AS ≤ 2 was compared to C and one GO MF gene set when RT-AS ≤ 2 was compared to RT-AS ≥ 10 with no other group comparisons showing differences in the tested gene sets or pathways. For blood samples sequenced at MGI subjected to GSEA (Fig. 1) no group comparison showed differences in any of the tested gene sets or pathways. Thereby, no gene sets or pathways were differentially expressed when both sequencing datasets were cross compared.

**Fig. 5:**
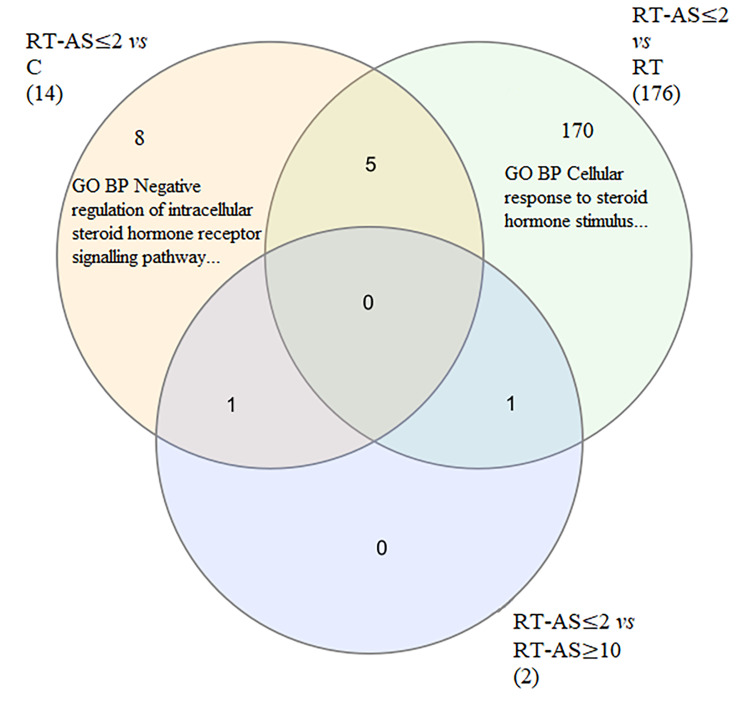
A Venn Diagram of GO BP gene sets that overlapped between the standard and CoolMPS sequencing datasets of the muscle samples, showing comparisons of RT-AS ≤ 2 (n = 15) to C (n = 5), RT (n = 17) and RT-AS ≥ 10 (n = 11), which were the only comparisons that had differences in GO BP gene sets. Numbers in brackets indicate the total number of differentially expressed GO BP gene sets for that comparison. C: non-resistance trained control group; RT: Resistance Trained control group; RT-AS ≤ 2: Resistance Trained participant who self-declared AAS exposure ceased ≤ 2 weeks before sampling; RT-AS ≥ 10: Resistance Trained participant who self-declared AAS exposure ceased ≥ 10 weeks before sampling

Overlapping differentially expressed gene sets/pathways from cross comparison of the muscle sequencing datasets are shown in Table 3. The greatest number of differentially expressed gene sets/pathways occurred when RT-AS ≤ 2 was compared to RT. All seventy-four (Table 3) of the differentially expressed Reactome pathways in this comparison were unique and eleven of twelve of the differentially expressed KEGG pathways for this comparison were unique. A Venn Diagram for RT-AS ≤ 2 compared to C, RT and RT-AS ≥ 10 for GO BP gene sets are shown in Fig. 5.

Lists of all gene sets and pathways and associated FDR values for all returning participant and group comparisons that were subjected to GSEA are available on OSF [35]. Lists of GO BP and GO MF gene sets and KEGG and Reactome pathways that have overlapping expression data with FDR < 0.05 based on cross comparison of the muscle sequencing datasets are also available (Additional File 4–7).

## Discussion

Although AAS are primarily used for hypertrophic benefit, their adverse and corollary effects on other physiological systems are also well documented [62]. AAS usage is known to stimulate erythropoiesis directly and EPO synthesis in the kidney [63], alongside promoting erythropoietic stem cell differentiation [64]. rHuEPO administration studies [12, 13] have shown a cross-platform [15] whole blood transcriptional signature to rHuEPO doping that may provide novel biomarkers for the haematological module of the ABP that are largely unconfounded by exercise [13] and altitude exposure [14]. Despite this connection, limited research [65] on the potential of a whole blood transcriptional response to AAS exposure exists, although one study has shown that circulating levels of the liver specific miR-122 may act as a biomarker of testosterone abuse [19]. In this present study, RNA libraries were sequenced twice and cross-compared to verify the identified differentially expressed genes/pathways. However, it should be noted that orthogonal method validation (e.g., qPCR) would provide a further level of verification to the findings in this study. Additionally, only DGE and GSEA was conducted on the sequencing datasets. Although not an intended goal of this research study, alternative splicing/isoform analysis may have identified additional differentially expressed transcripts that do not emerge at the gene level and future studies may want to investigate these transcripts. AAS users who self-declared AAS cessation ≤ 2-weeks prior to first sampling and returned 19–28 weeks later, did not show differences in gene expression in whole blood between time points when all samples (i.e., the full dataset) was analysed (Additional File 2 Table 14a). Re-sequencing of these RNA libraries and removal of one sample for aberrant sequencing quality control values unique to this re-sequencing dataset (i.e., the MGI dataset) did show some differences in gene expression between time points (Additional File 2 Table 14b), but this reduction in statistical power from lower participant numbers could have influenced this dataset specific result. Both returning participant whole blood sequencing datasets did not show any differences in the gene sets or pathways tested between timepoints.

Cross-comparison of both whole blood sequencing datasets of group comparisons showed that RT-AS ≤ 2 did not differ in gene expression to C, RT, and RT-AS ≥ 10. Both sequencing datasets did show RT-AS ≥ 10 did have had one gene (*IGLV3-10*) upregulated when compared to RT. However, it would be difficult to conclude that this is a lingering whole blood transcriptional biomarker of AAS usage given there was no differences in this gene in users who ceased AAS exposure at an earlier time frame, or controls. Additionally, this gene codes for a variable domain of an immunoglobulin light chain and is likely to be impacted heavily by the immune response to pathogens/exogenous factors. Cross-comparison of both whole blood sequencing datasets for group comparisons did not reveal any differentially expressed gene sets or pathways.

For the two muscle sequencing datasets, cross-comparison of the paired sample analysis of returning participants post AAS exposure found that one gene (*CHRDL1*) was upregulated in visit two in both datasets (Table 2). *CHRDL1* encodes for the Chordin-Like 1 (CHRDL1) protein which is a known antagonist of bone morphogenetic protein (BMP), and BMP signalling is known to play a key role in muscle development, hypertrophy and regeneration [66]. In adult mice, in the absence of injury, increasing BMP expression, or BMP receptor activity, is known to induce hypertrophy via activation of mTOR signalling [67]. Furthermore, inhibition of BMP signalling causes muscle atrophy and abolishes the hypertrophic phenotype of myostatin-deficient mice, with BMP signalling being regarded as a fundamental hypertrophic signal in mice [68]. In the present study, these three returning participants (RP2-4) that showed an upregulation of *CHRDL1* in their second visit, also showed a 4.4 ± 0.3 kg loss of Fat Free Mass as measured with bioelectrical impedance [21], a finding which corroborates with CHRDL1 as an antagonist of BMP and BMP inhibition causing atrophy. Furthermore, RP2 also exhibited a decrease in muscle fibre cross-sectional area (CSA) between visits (7854 vs. 5677 µm^2^), whereas for RP3 an increase in CSA (7167 vs. 7889 µm^2^) was observed and RP4 did not have a sample stored for immunohistochemistry on first sample visit [21]. When cross-compared, none of the tested gene sets or pathways showed differences in both muscle sequencing datasets for comparisons of returning participant visits.

Cross-comparison, of both muscle sequencing datasets showed that the greatest number of differentially expressed genes occurred when RT-AS ≤ 2 was compared to other groups (Table 2). Comparing RT vs. C (the effect of training), RT-AS ≤ 2 vs. C (the effect of training and acute AAS usage) and RT-AS ≤ 2 vs. RT (the effect of acute AAS usage), showed that nine differentially expressed genes (*ABCA7, ARHGEF17, BOK, FILIP1L, LDAF1, RBL1, RPIA, SDC4, ZFP36L1*) overlap (Fig. 2) between RT-AS ≤ 2 vs. C and RT-AS ≤ 2 vs. RT, but were not differentially expressed in RT vs. C, potentially indicating this is caused by acute AAS usage and not from training alone. Amongst these genes, associated with performance benefit would be a downregulation of *RBL1*, with reduced expression of this transcriptional corepressor being associated with mitochondrial biogenesis, typically stimulated by exercise [69]. However, contradictory to performance/hypertrophic benefit amongst these genes was downregulation of *SDC4*, a proteoglycan known to be crucial for muscle differentiation [70] that may act as a reservoir for promyostatin [71], subsequently inhibiting the formation of active myostatin, with reduced expression being associated with elevated levels of myostatin [71]. Additionally, *BOK*, a pro-apoptotic member of the BCL-2 family, was upregulated, with this family of proteins being upregulated in denervation-induced muscle atrophy [72] and *ZFP36L1* was downregulated, with reduced expression being associated with reduced skeletal muscle mass and reduced satellite cell numbers [73].

Although two genes (*NAP1L4* and *CARS1*) were differentially expressed when RT-AS ≥ 10 was compared to C (Table 2) in both muscle sequencing datasets, these two genes were not unique to this comparison and were also differentially expressed in RT vs. C and RT-AS ≤ 2 vs. C (Fig. 2) indicating they are unlikely to be unique markers of long-term steroid usage and more likely due to resistance training alone as they were also not differentially expressed when RT-AS ≤ 2 was compared to RT.

Hierarchical clustering and heatmaps of muscle samples using the top 30 most significantly differentially expressed genes by FDR, with a minimum 1.2-fold-change, for the group comparison RT-AS ≤ 2 to RT showed that most samples within RT-AS ≤ 2 clustered together in both sequencing datasets (Figs. 3 and 4). However, previous research in animal husbandry has shown that similar hierarchical clustering methods using 20 differentially expressed genes in liver samples can fully distinguish boars and calves treated with AAS with no cross-group clustering [20]. Using an Orthogonal Projections to Latent Structures Discriminant Analysis (OPLS-DA) model [74], proteomic analysis of human vastus lateralis muscle samples from 10 current AAS users, who had used large AAS doses (> 800 mg) for 5–15 years, showed a clear separation from 7 non-AAS using resistance trained controls. Liquid chromatography followed by tandem spectrometry identified 14 protein spots (representing nine different proteins) of significant difference in relative quantity between the doped and clean groups [74]. However, analysis of the RNA-Seq data from both muscle datasets in this study did not identify any of the genes that correspond to these nine proteins as differentially expressed (Additional File 3) in any comparison. The participants in this present study having much lower AAS exposure regimens could have contributed to this discrepancy.

Cross-comparison of both muscle sequencing datasets showed that no gene sets or pathways were differentially expressed when RT was compared to C, with differences only observed when RT-AS ≤ 2 was compared to C, RT and RT-AS ≥ 10 with no differences between returning participants visits (Table 3). Notably, eight GO BP gene sets (Fig. 5), were uniquely differentially expressed for the comparison RT-AS ≤ 2 vs. C, including a downregulated of a gene set that reduces the activity of intracellular steroid hormone receptor signalling pathways, corroborating with the known AR pathway for AAS induced transcription. For GO MF gene sets, none were uniquely differentially expressed for the comparison RT-AS ≤ 2 vs. C (Additional File 5). Two KEGG Pathways were differentially expressed when RT-AS ≥ 10 was compared to RT, but no KEGG Pathways were differentially expressed when RT-AS ≥ 10 was compared to C, making it unlikely that these are long-term markers of AAS usage (Table 3).

This study has numerous methodological limitations, of which some have been detailed elsewhere [21]. Due to the known deleterious impact of AAS on health markers [62] it can be argued that the only ethically feasible way to study high-dose/sustained AAS usage is through observational research [75]. Inherently this results in confounding variables that could impact the results of this study including: AAS regimens and date of cessation differing between participants, self-reported AAS cycles being fallible to recall errors, reported time frames of AAS abstinence being inaccurate and AAS quality being unknown. The AAS exposure results in this study therefore only serve as estimates and for RT-AS ≥ 10 time since last AAS cessation had a large range. Despite these inaccuracies there is some value in obtaining verbal declarations of AAS usage as it enables a broad classification between “high” and “low” doses as previous research has identified that reported cycles from 100 AAS users varied 10-fold in maximum weekly dosage and 100-fold in cumulative cycle dose [76]. Although most participants in RT and RT-AS ≤ 2 were recreational lifters [21], training regimens and training volumes will differ amongst participants, with this being a notable confounding variable that could influence differences in expressed genes. Differences in age and other lifestyle factors (e.g., dietary habits) are other possible confounding variables. The number of participants within RT-AS who returned for sampling post AAS exposure was low, but only six participants verbalized intentions for complete removal of AAS for ≥ 18 weeks after usage and only five were sampled on a second visit. It is common for AAS users to undergo a “blast and cruise” usage pattern [77] in which AAS exposure peaks (the “blast”) but then never drops to genuine physiological levels of testosterone where users “cruise” on above physiological testosterone levels instead of AAS cessation or using true TRT. Finding AAS users who did not partake in a “blast and cruise” usage pattern, which heavily confounds AAS cessation post initial exposure, was difficult and contributed to the low number of returning participants. Group C also had the lowest number of recruited participants; however this was a difficult group to recruit as individuals who do not resistance train were not as interested to partake in a study that did not offer remuneration, unlike those in Group RT and RT-AS. Future studies investigating the impact of AAS usage on gene expression should focus on sampling higher numbers of AAS users longitudinally, ideally pre, during and post AAS exposure, as paired-sample analysis reduces the impact of confounding variables. AAS samples could also be collected and tested for purity. Future studies could focus on AAS administration to cell culture lines to further investigate differentially expressed genes identified in this study. Serum and plasma samples were also collected from participants and a future avenue of research could be to investigate if an AAS metabolomic doping signature could be identified, similar to how a rHuEPO metabolomic doping signature has been identified [78].

## Conclusions

In conclusion, although the observational nature of this study would have impacted its findings, given that no differentially expressed genes were identified in whole blood in both sequencing datasets when RT-AS ≤ 2 was compared to RT or C, this current data suggests that it seems unlikely that a whole blood transcriptional signature could be used to identify AAS doping. However, in muscle, AAS exposure had a greater impact on gene expression, with differential expression in genes known to impact hypertrophic processes. Furthermore, the majority of current AAS users clustered together in muscle gene expression profiles, showing that a subset of genes seems to be both up- and downregulated from AAS exposure, with this finding potentially contributing to furthering our understanding of AAS induced hypertrophic processes.

## Electronic supplementary material

Below is the link to the electronic supplementary material.

Supplementary Material 1

Supplementary Material 2

Supplementary Material 3

Supplementary Material 4

Supplementary Material 5

Supplementary Material 6

Supplementary Material 7

## Data Availability

Group/returning participant information matrices used in edgeR for DGE analysis, raw salmon count matrices (quant files) and R code used for DGE analysis and GSEA analysis are publicly available on OSF [https://osf.io/27rjy/]. Additionally, FastQC per base sequence quality scores and interactive MultiQC reports for FastQ Screen and RSeQC are publicly available on OSF [https://osf.io/27rjy/]. Raw FASTQ files have not been made publicly available due to the remote possiblity of participant anonymity being compromised but are available from the corresponding author on reasonable request.

## List of abbreviations

AAS: Anabolic Androgenic Steroid
ABP: Athlete Biological Passport
AR: Androgen Receptor
BMP: Bone Morphogenetic Protein
C: Control Group (non-resistance trained)
DGE: Differential Gene Expression
DNB: DNA nanoballs
EPO: Erythropoietin
FDR: False Discovery Rate
GO BP: Gene Ontology Biological Processes
GO MF: Gene Ontology Molecular Functions
GSEA: Gene Set Enrichment Analysis
KEGG: Kyoto Encyclopaedia of Genes and Genomes
mTOR: Mammalian Target of Rapamycin
OSF: Open Science Framework
PCT: Post Cycle Therapy
PREV: Participants who ceased AAS exposure ≥ 52 weeks ago
RP: Returning Participant (i.e., a participant in RT-AS who was sampled once and then returned for a second sampling visit)
RT: Resistance Trained Group
RT-AS ≤ 2: Resistance trained current AAS users who ceased exposure ≤ 2 weeks prior to sampling
RT-AS ≥ 10: Resistance trained current AAS users who ceased exposure ≥ 10 weeks prior to sampling
rHuEPO: Recombinant Human Erythropoietin
TRT: Testosterone Replacement Therapy
UoB: University of Brighton
