# Supplementary material for: An observational human study investigating the effect of anabolic androgenic steroid use on the transcriptome of skeletal muscle and whole blood using RNA-Seq
Source: BMC Med Genomics. 2023 May 3;16:94. doi: 10.1186/s12920-023-01512-z (PMC10157927; doi:10.1186/s12920-023-01512-z)
Supplement: Supplementary file 1 — Supplementary Material 1 [file 12920_2023_1512_MOESM1_ESM.pdf]

Additional File 1 – MDS & PCA Plots

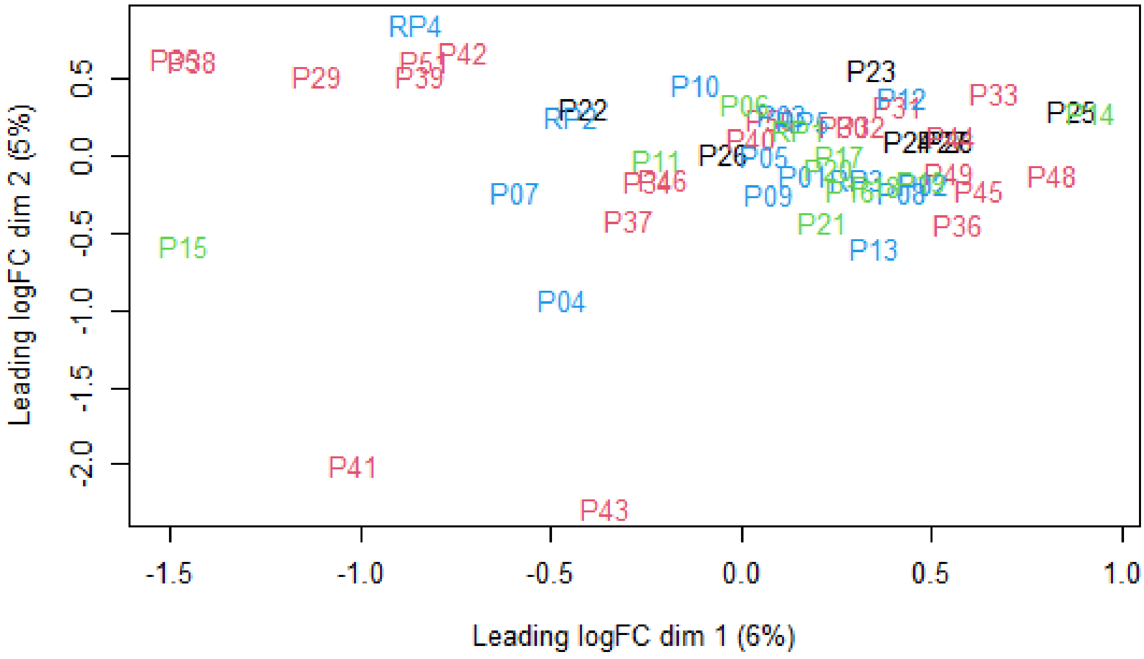

Additional File 1 Figure 1a. MDS Plot of blood samples (n=55) to be used in group comparisons sequenced at the University of Brighton with standard chemistry reagents from Group C (black, n=7), Group RT (red, n=22), Group RT-AS ≤ 2 (blue, n=15) and Group RT-AS ≥ 10 (green, n=11).

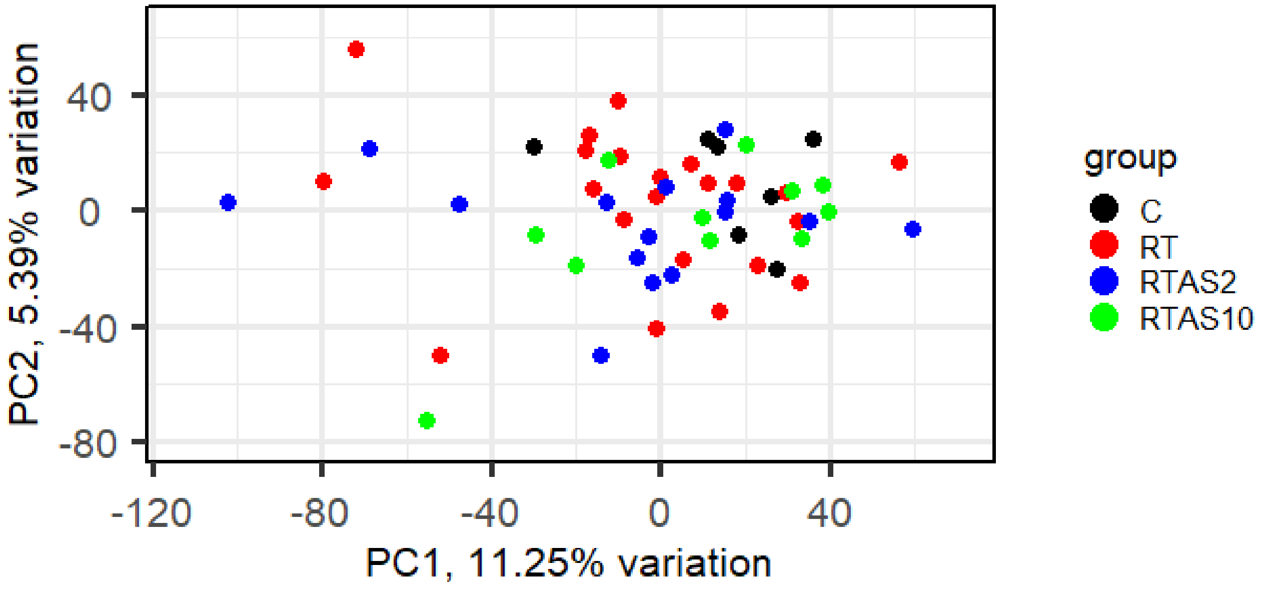

Additional File 1 Figure 1b. PCA Plot of blood samples (n=55) to be used in group comparisons sequenced at the University of Brighton with standard chemistry reagents from Group C (n=7), Group RT (n=22), Group RT-AS ≤ 2 (n=15) and Group RT-AS ≥ 10 (n=11).

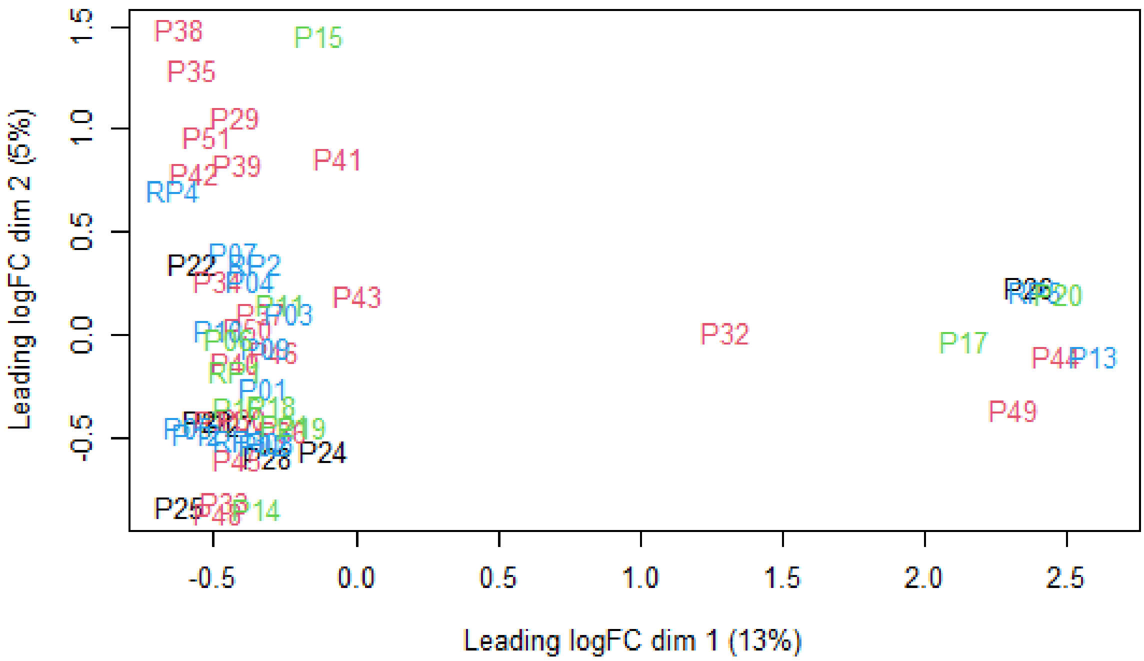

Additional File 1 Figure 2a. MDS Plot of blood samples (n=55) to be used in group comparisons sequenced at MGI with standard chemistry reagents from Group C (black, n=7), Group RT (red, n=22), Group RT-AS ≤ 2 (blue, n=15) and Group RT-AS ≥ 10 (green, n=11).

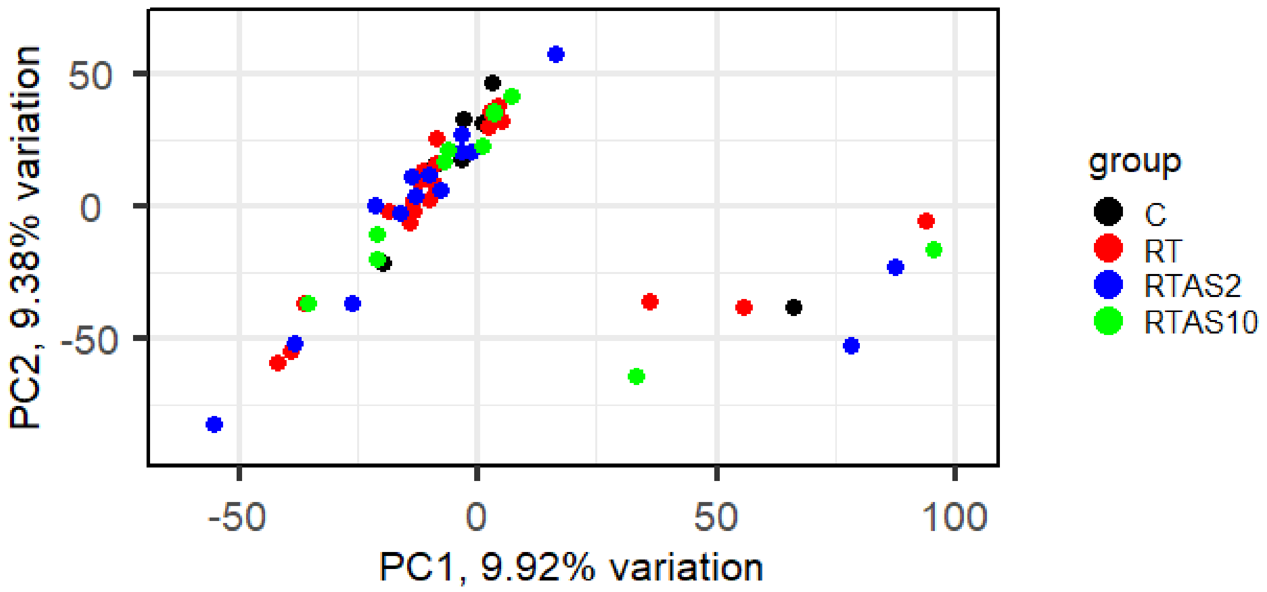

Additional File 1 Figure 2b. PCA Plot of blood samples (n=55) to be used in group comparisons sequenced at MGI with standard chemistry reagents from Group C (n=7), Group RT (n=22), Group RT-AS ≤ 2 (n=15) and Group RT-AS ≥ 10 (n=11).

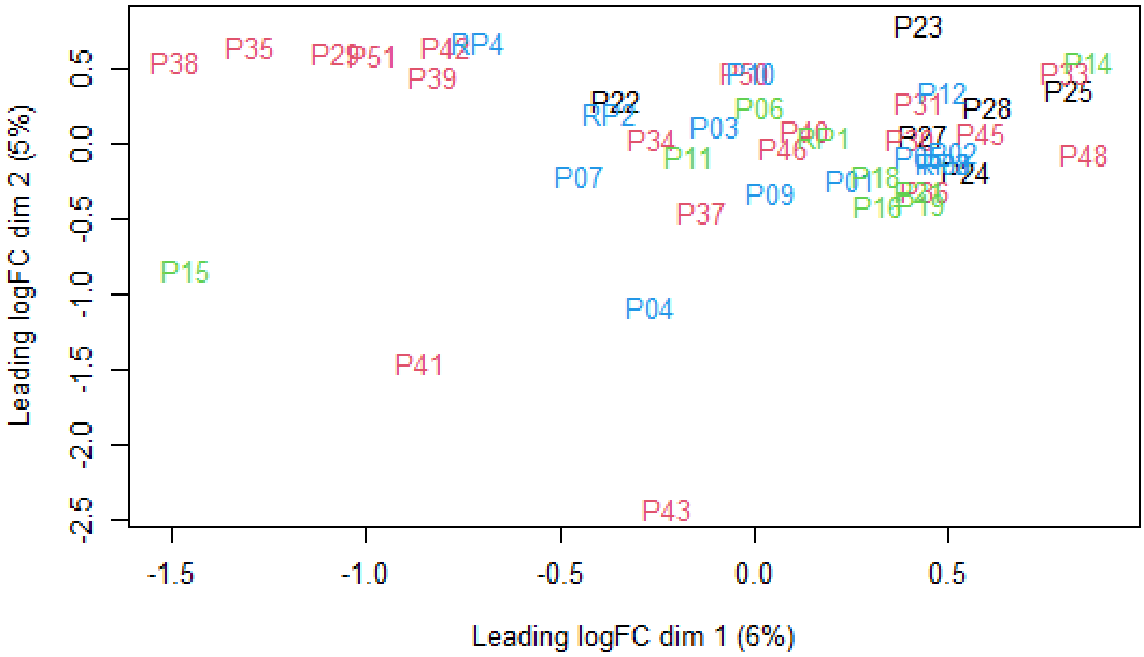

Additional File 1 Figure 3a. MDS Plot of blood samples (n=47) to be used in group comparisons sequenced at MGI with standard chemistry reagents after samples (n=8) sequenced on Flow Cell B Lane 1 were removed as outliers: Group C (black, n=6), Group RT (red, n=19), Group RT-AS ≤ 2 (blue, n=13) and Group RT-AS ≥ 10 (green, n=9).

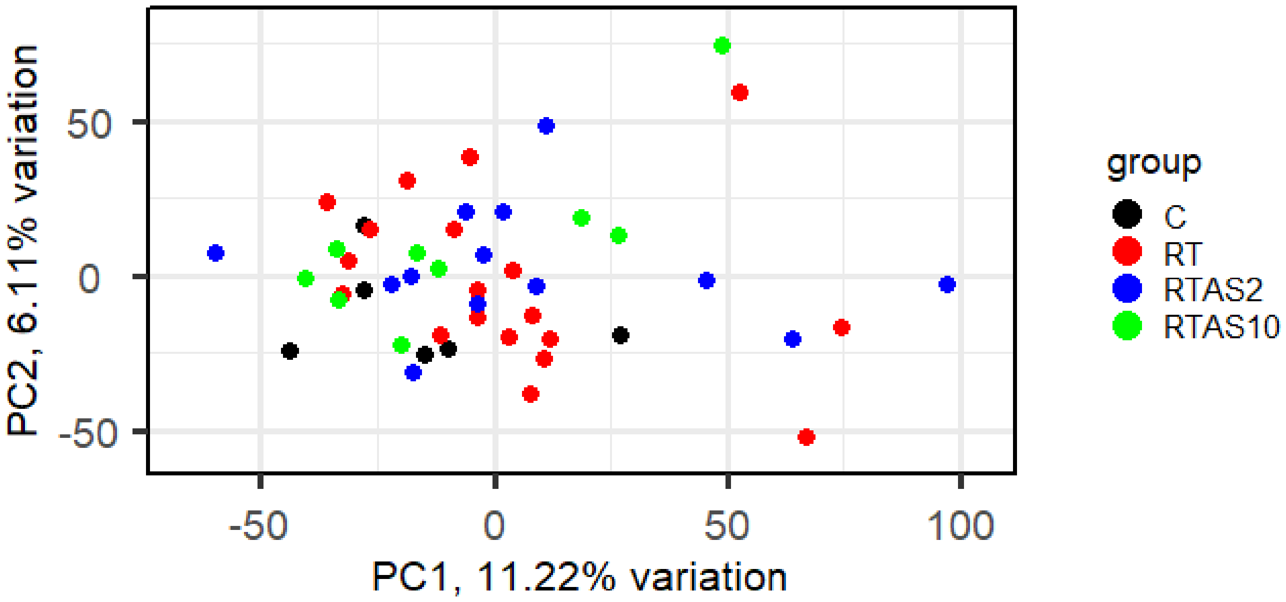

Additional File 1 Figure 3b. PCA Plot of blood samples (n=47) to be used in group comparisons sequenced at MGI with standard chemistry reagents after samples (n=8) sequenced on Flow Cell B Lane 1 were removed as outliers: Group C (n=6), Group RT (n=19), Group RT-AS ≤ 2 (n=13) and Group RT-AS ≥ 10 (n=9).

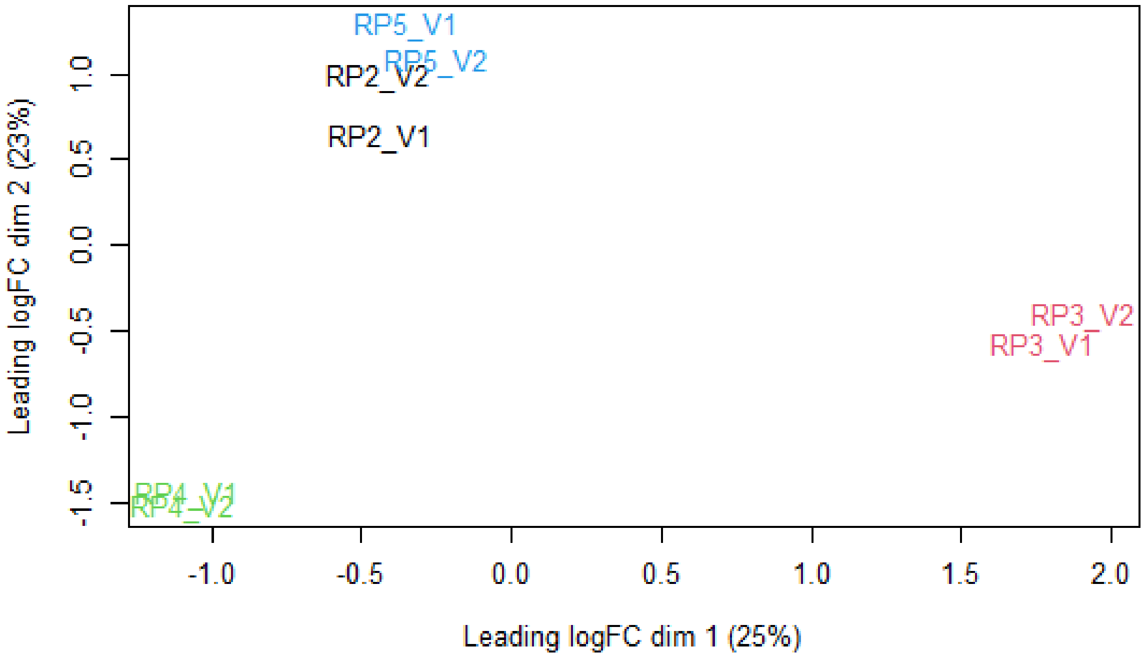

Additional File 1 Figure 4a. MDS Plot of blood samples (n=8) sequenced at the University of Brighton with standard chemistry reagents from Returning Participant (RP2-5) first and second visits (_V1 or _V2).

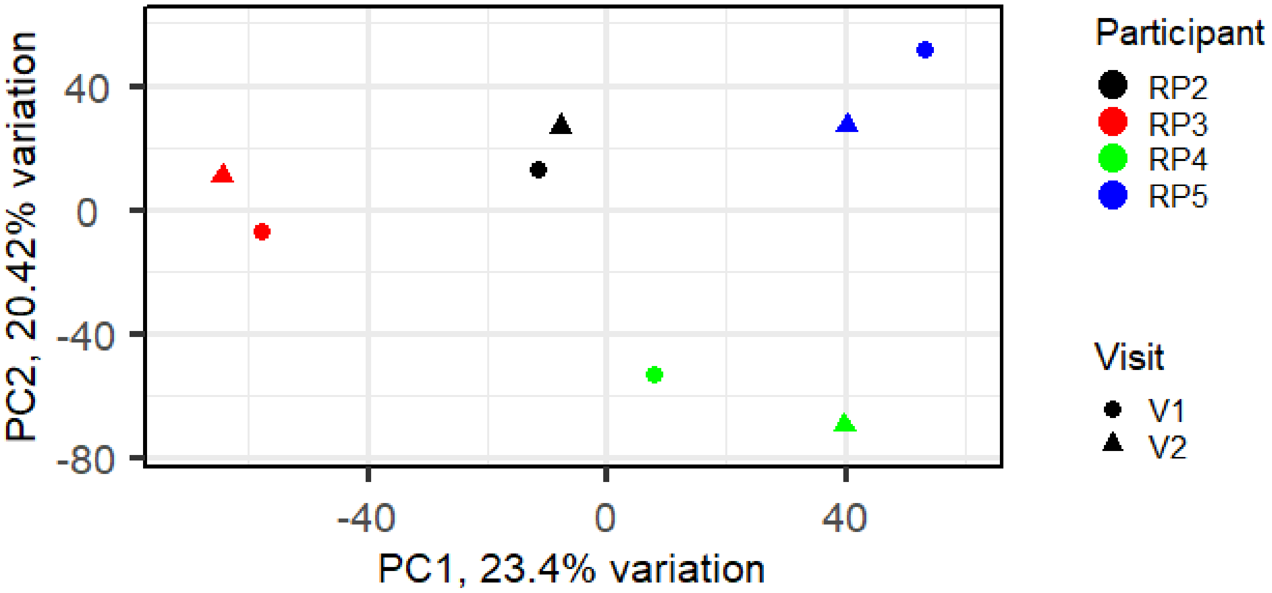

Additional File 1 Figure 4b. PCA Plot of blood samples (n=8) sequenced at the University of Brighton with standard chemistry reagents from Returning Participant (RP2-5) first and second visits (V1 or V2).

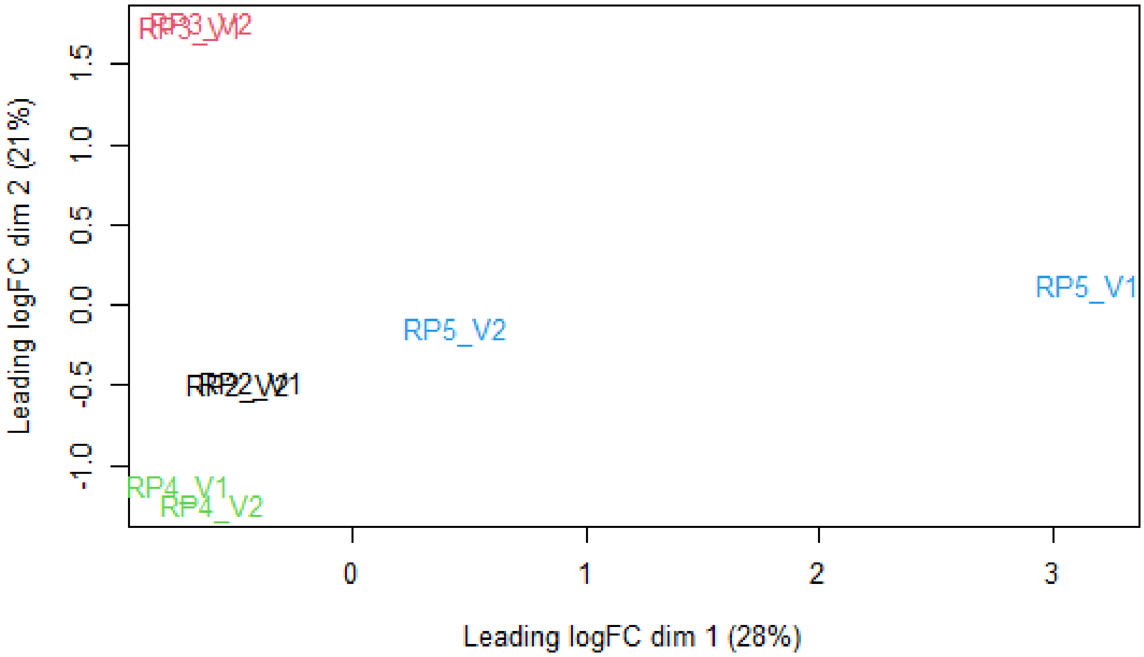

Additional File 1 Figure 5a. MDS Plot of blood samples (n=8) sequenced at MGI with standard chemistry reagents from Returning Participant (RP2-5) first and second visits (_V1 or _V2).

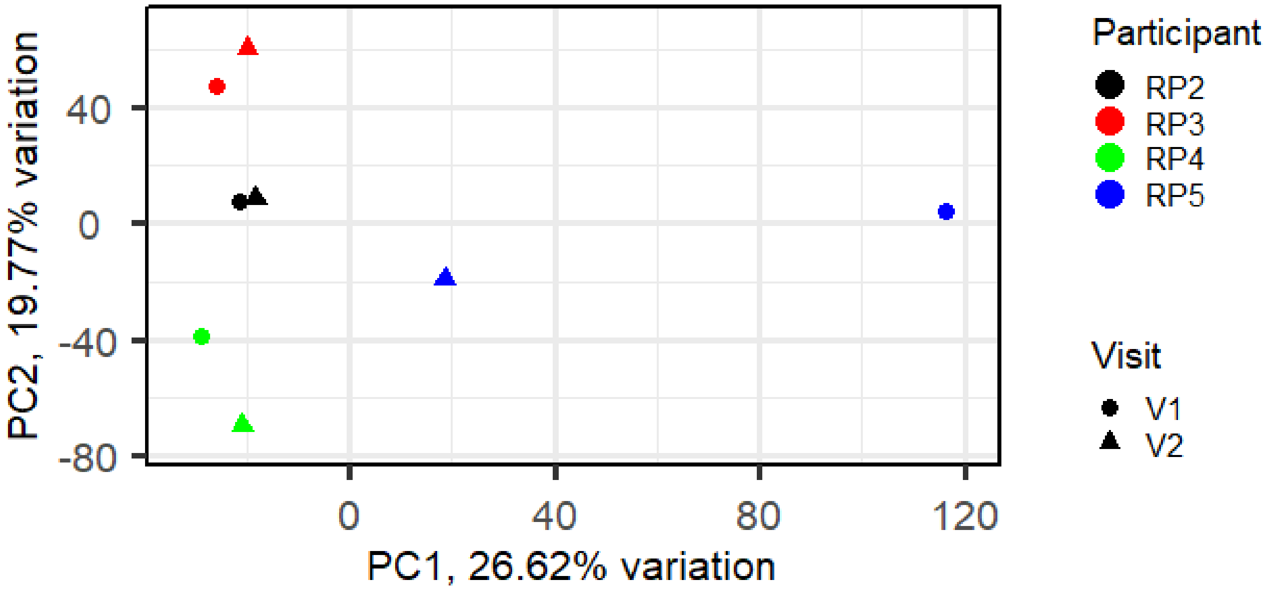

Additional File 1 Figure 5b. PCA Plot of blood samples (n=8) sequenced at MGI with standard chemistry reagents from Returning Participant (RP2-5) first and second visits (V1 or V2).

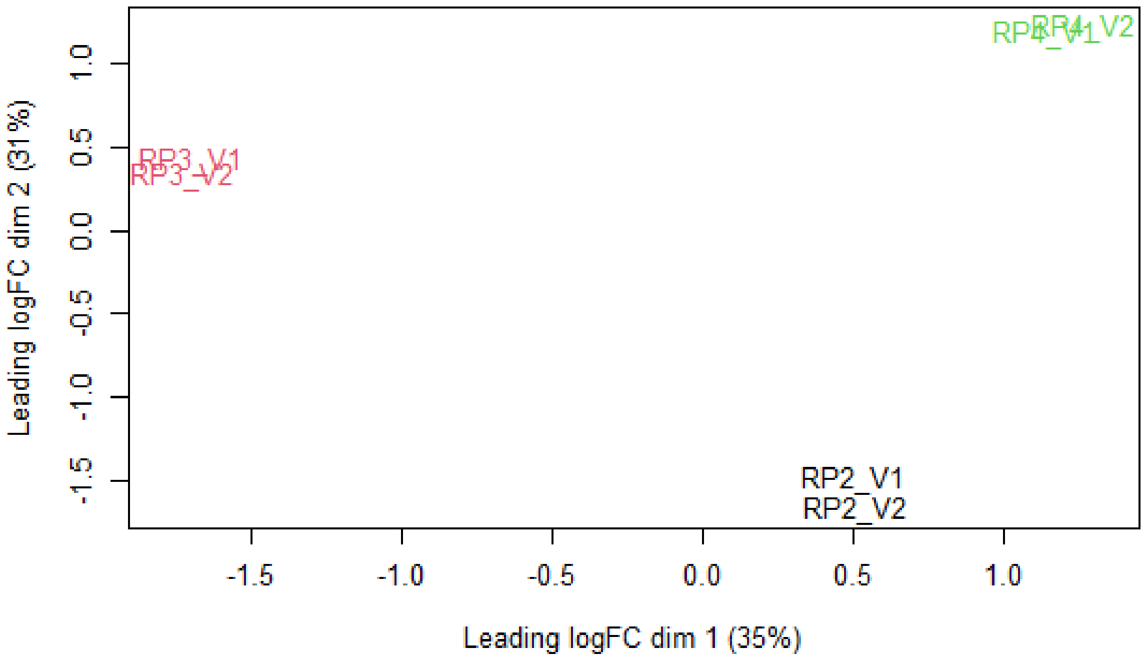

Additional File 1 Figure 6a. MDS Plot of blood samples (n=6) sequenced at MGI with standard chemistry reagents that underwent differential gene expression analysis from Returning Participant (RP2-4) first and second visits (_V1 or _V2).

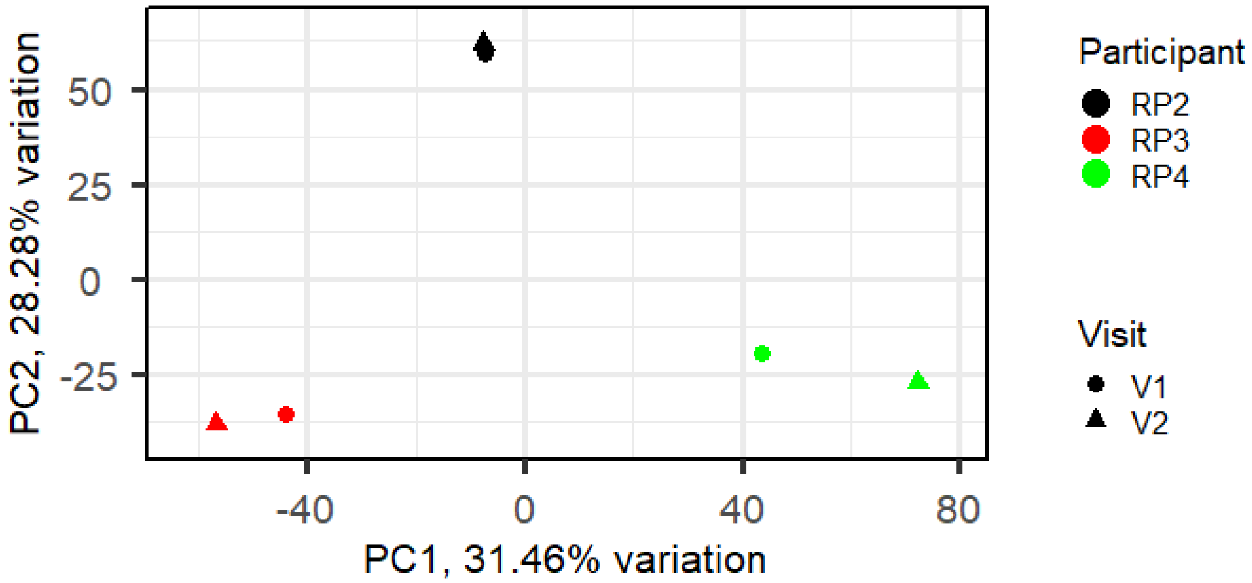

Additional File 1 Figure 6b. PCA Plot of blood samples (n=6) sequenced at MGI with standard chemistry reagents that underwent differential gene expression analysis from Returning Participant (RP2-4) first and second visits (V1 or V2).

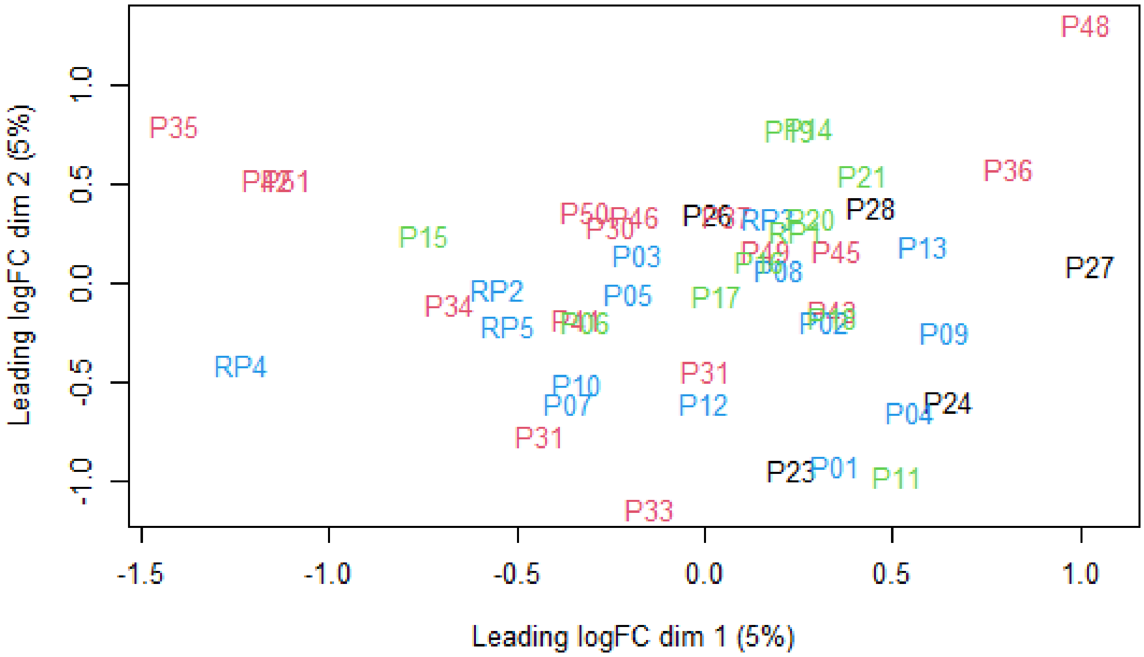

Additional File 1 Figure 7a. MDS Plot of muscle samples (n=48) to be used in group comparisons sequenced with MGI standard chemistry reagents at MGI from Group C (black, n=5), Group RT (red, n=17), Group RT-AS ≤ 2 (blue, n=15) and Group RT-AS ≥ 10 (green, n=11) subjected to Differential Gene Expression analysis.

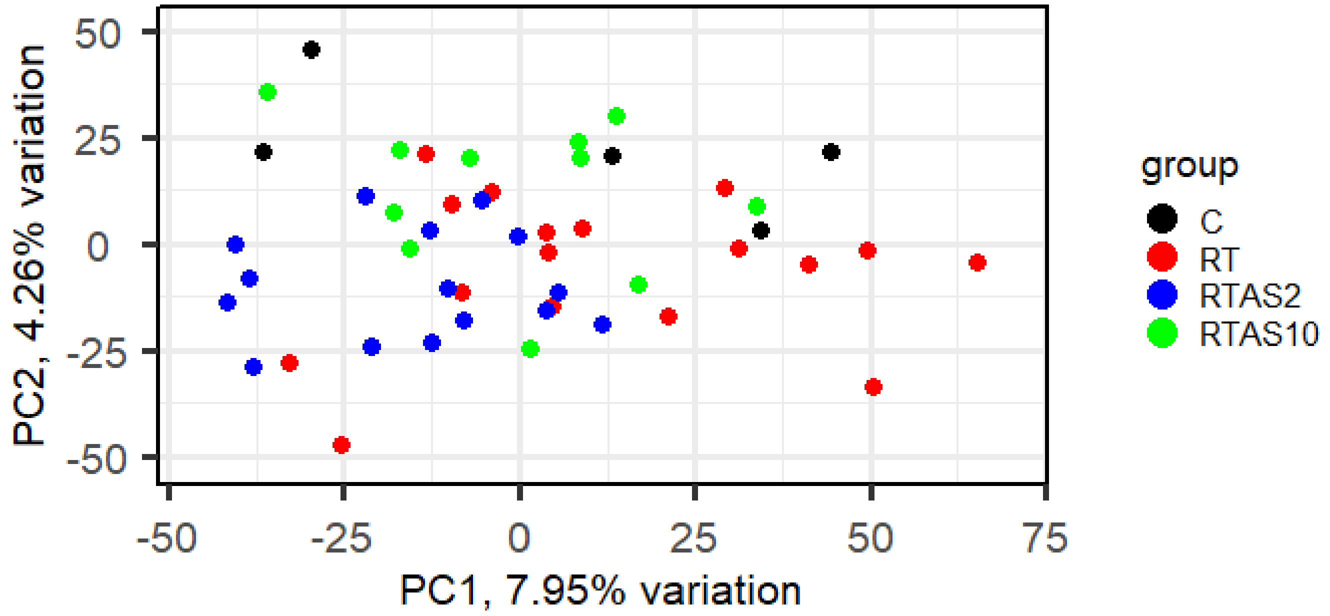

Additional File 1 Figure 7b. PCA Plot of muscle samples (n=48) to be used in group comparisons sequenced with MGI standard chemistry reagents at MGI from Group C (n=5), Group RT (n=17), Group RT-AS ≤ 2 (n=15) and Group RT-AS ≥ 10 (n=11) subjected to Differential Gene Expression analysis.

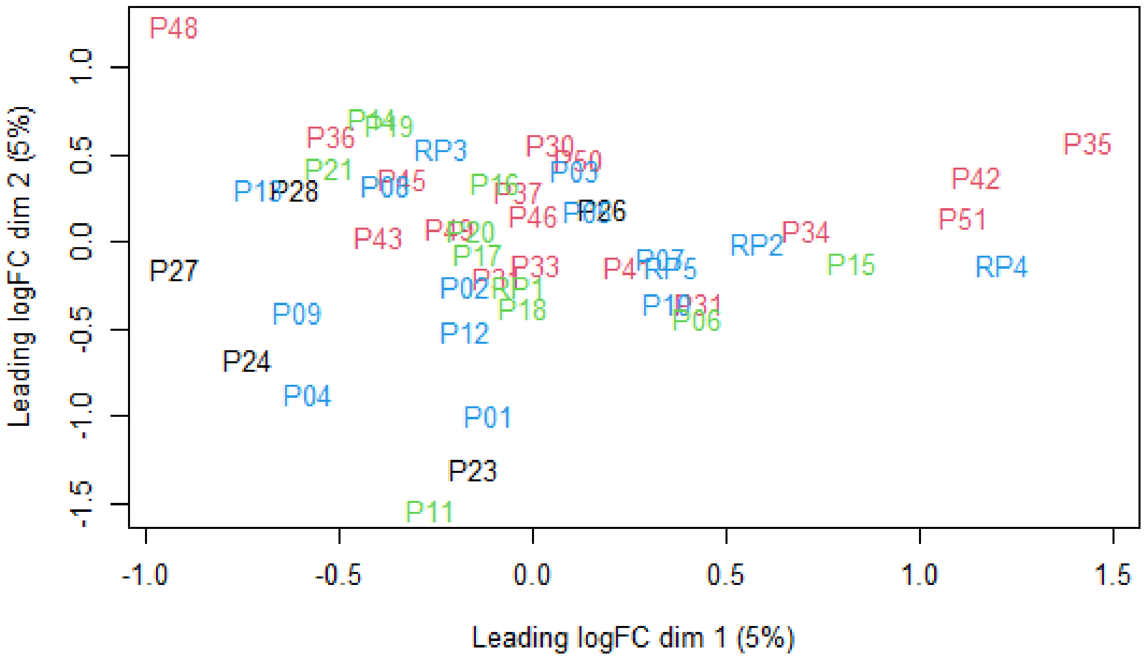

Additional File 1 Figure 8a. MDS Plot of muscle samples (n=48) to be used in group comparisons sequenced with CoolMPS reagents at MGI from Group C (black, n=5), Group RT (red, n=17), Group RT-AS ≤ 2 (blue, n=15) and Group RT-AS ≥ 10 (green, n=11) subjected to Differential Gene Expression analysis.

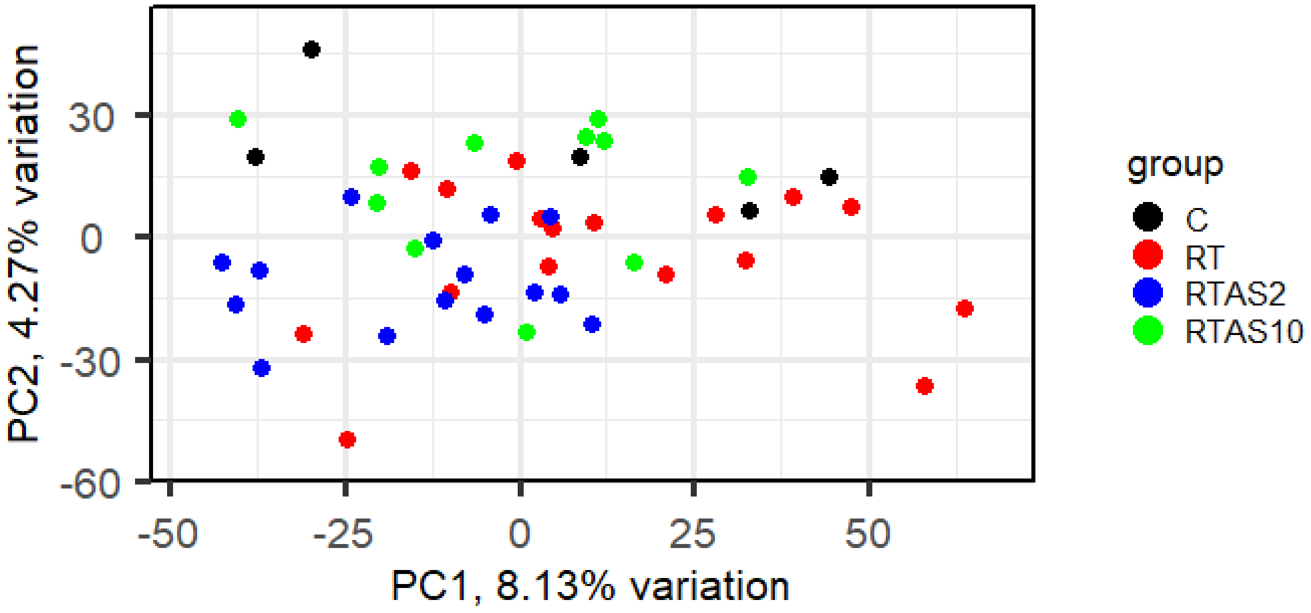

Additional File 1 Figure 8b. PCA Plot of muscle samples (n=48) to be used in group comparisons sequenced with CoolMPS reagents at MGI from Group C (n=5), Group RT (n=17), Group RT-AS ≤ 2 (n=15) and Group RT-AS ≥ 10 (n=11) subjected to Differential Gene Expression analysis.

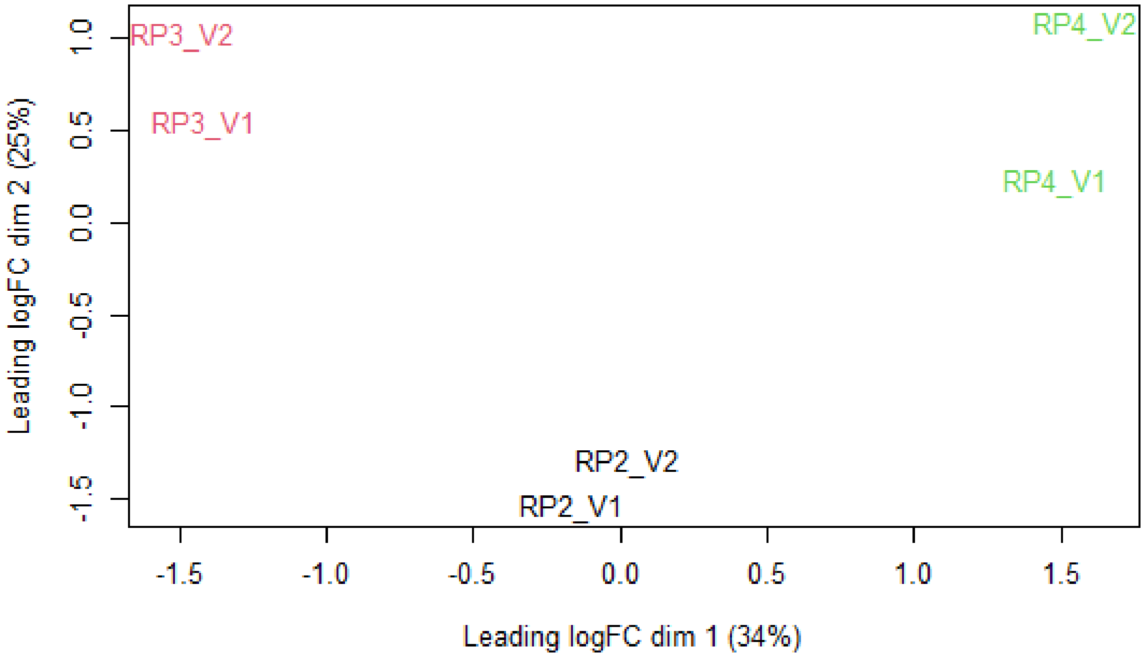

Additional File 1 Figure 9a. MDS Plot of muscle samples (n=6) sequenced with Standard MGI reagents at MGI from Returning Participant (RP2-4) first and second visits (_V1 or _V2).

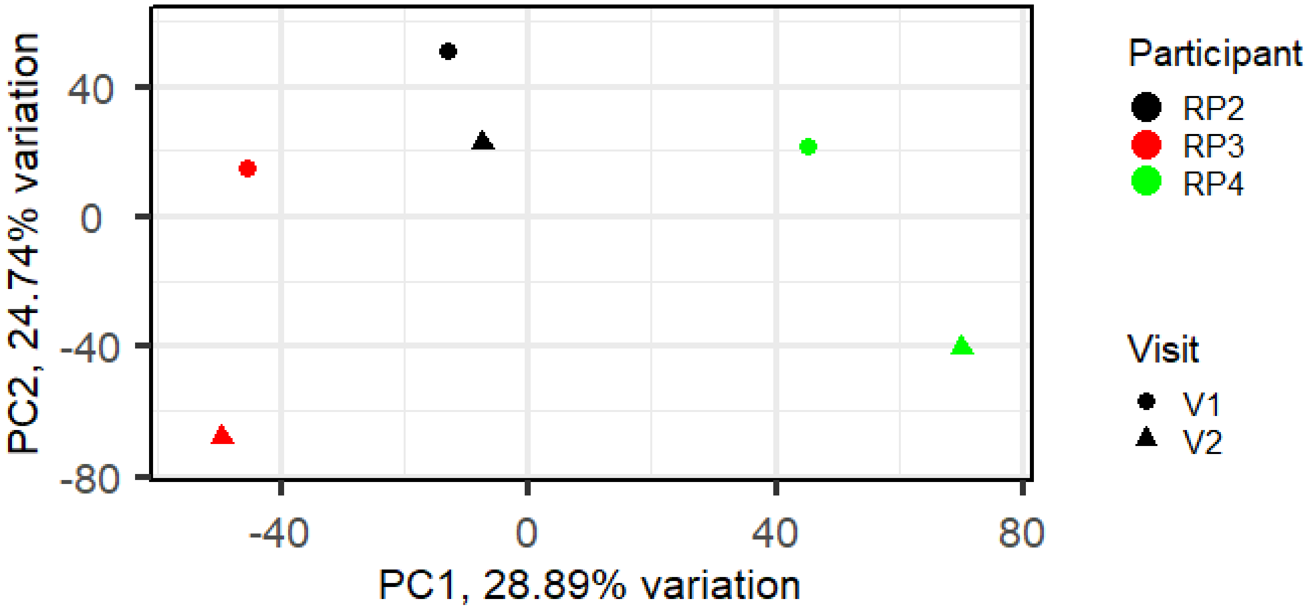

Additional File 1 Figure 9b. PCA Plot of muscle samples (n=6) sequenced with Standard MGI reagents at MGI from Returning Participant (RP2-4) first and second visits (V1 or V2).

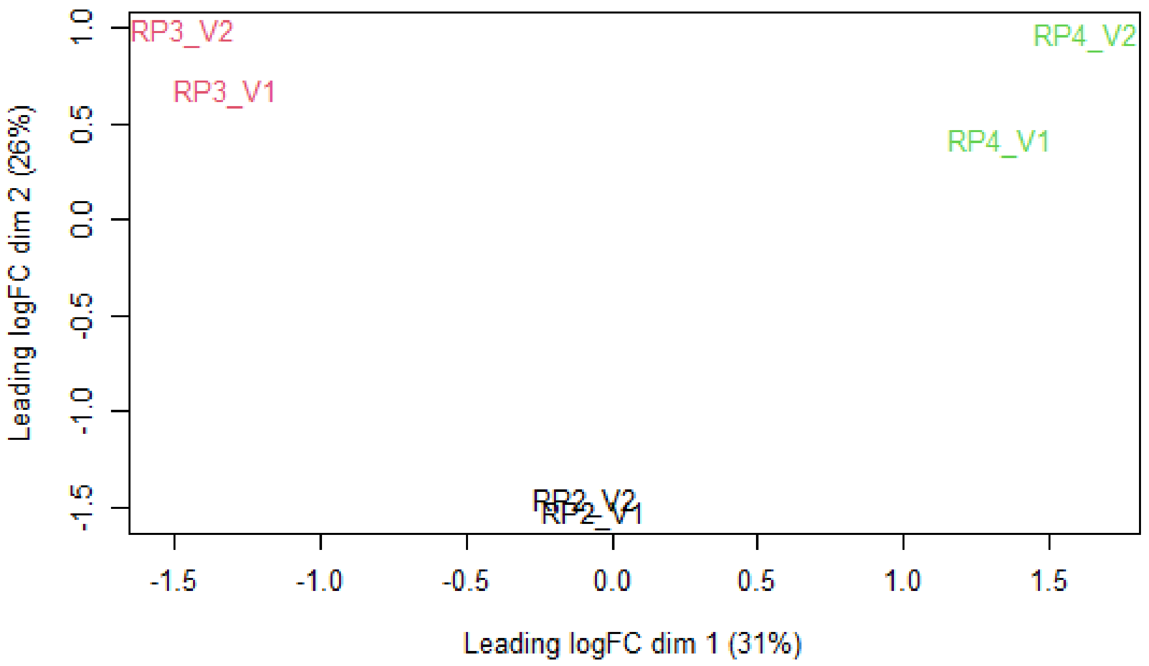

Additional File 1 Figure 10a. MDS Plot of muscle samples (n=6) sequenced with CoolMPS reagents at MGI from Returning Participant (RP2-4) first and second visits (_V1 or _V2).

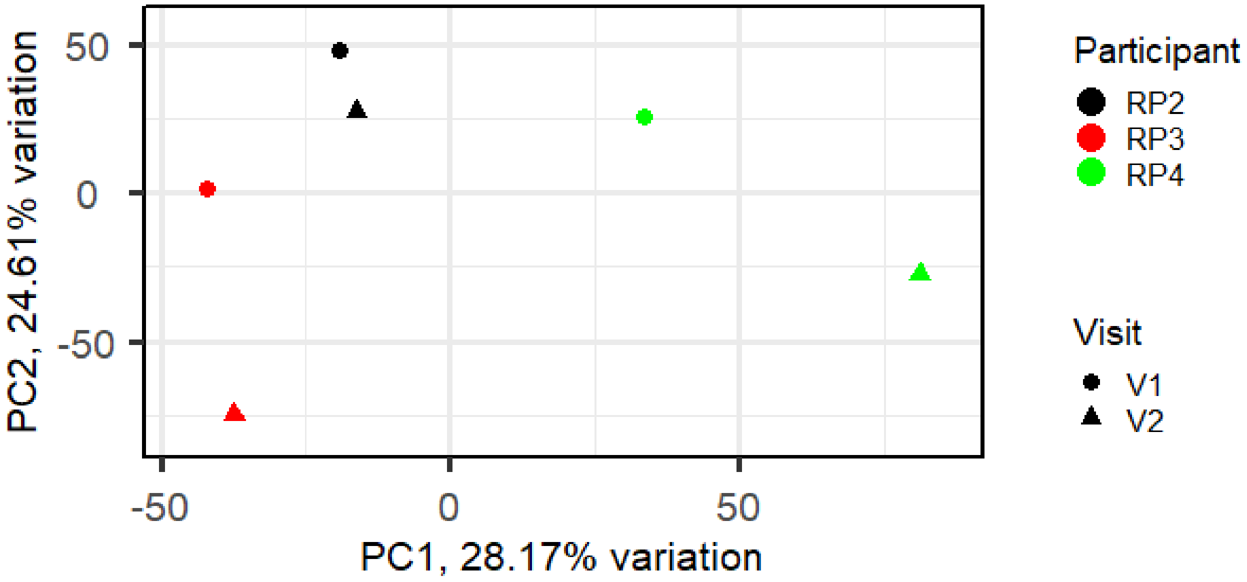

Additional File 1 Figure 10b. PCA Plot of muscle samples (n=6) sequenced with CoolMPS reagents at MGI from Returning Participant (RP2-4) first and second visits (V1 or V2).

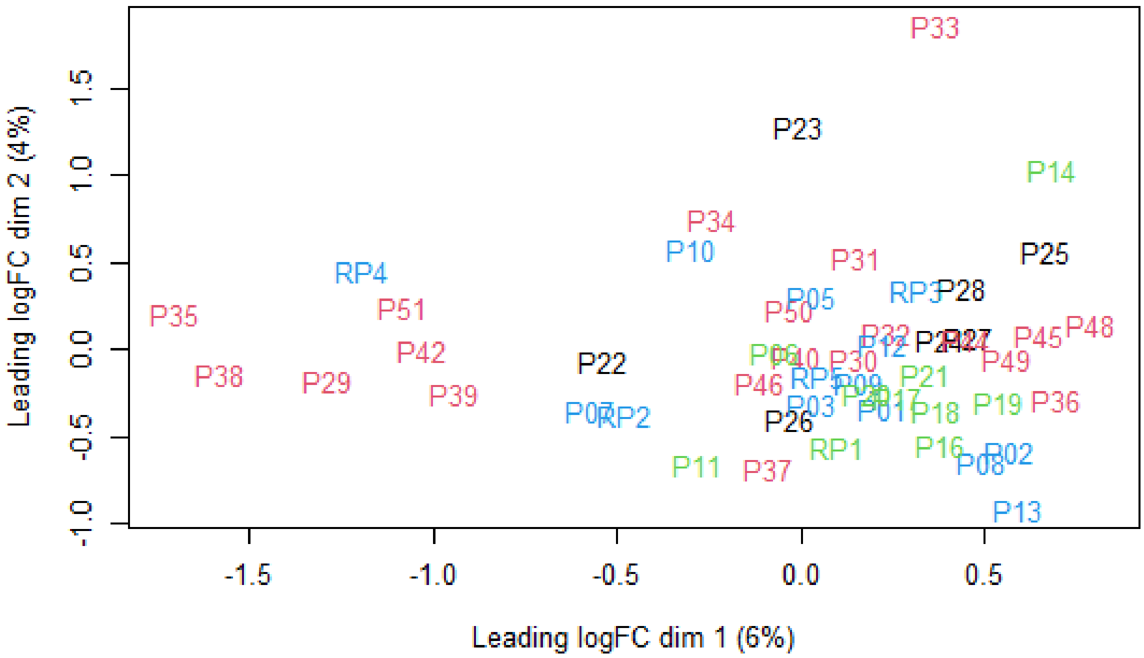

Additional File 1 Figure 11a. MDS Plot of blood samples (n=51) sequenced at the University of Brighton with standard chemistry reagents that underwent differential gene expression analysis. Group C (black, n=7), Group RT (red, n=20), Group RT-AS ≤ 2 (blue, n=14) and Group RT-AS ≥ 10 (green, n=10).

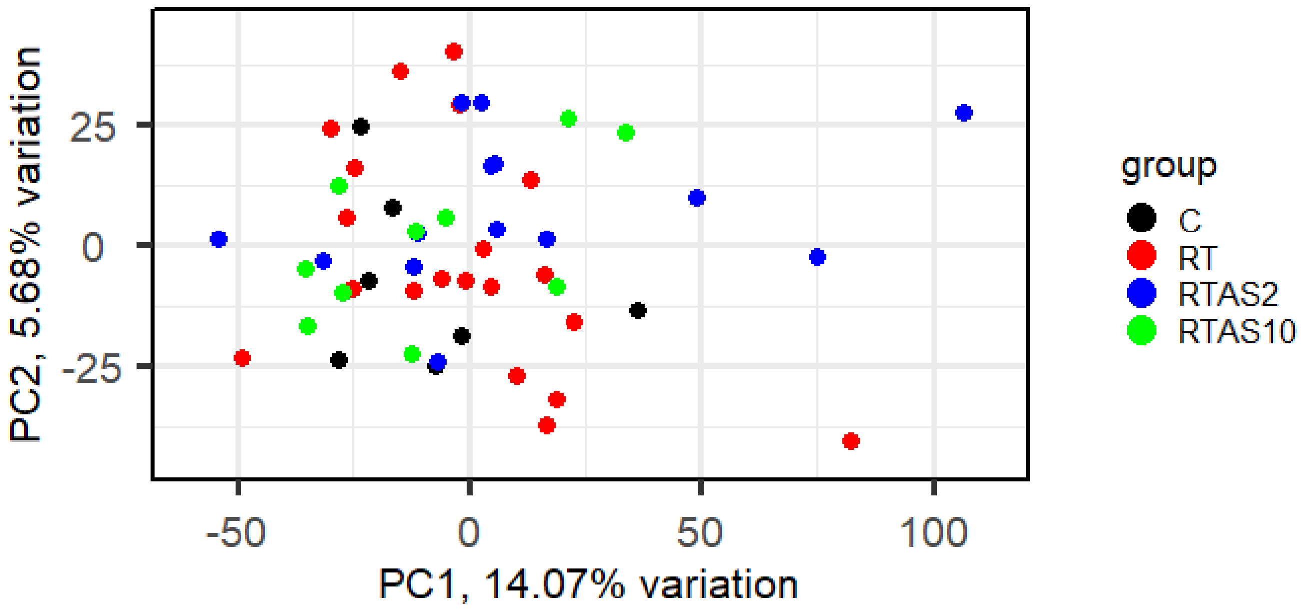

Additional File 1 Figure 11b. MDS Plot of blood samples (n=51) sequenced at the University of Brighton with standard chemistry reagents that underwent differential gene expression analysis. Group C (n=7), Group RT (n=20), Group RT-AS ≤ 2 (n=14) and Group RT-AS ≥ 10 (n=10).

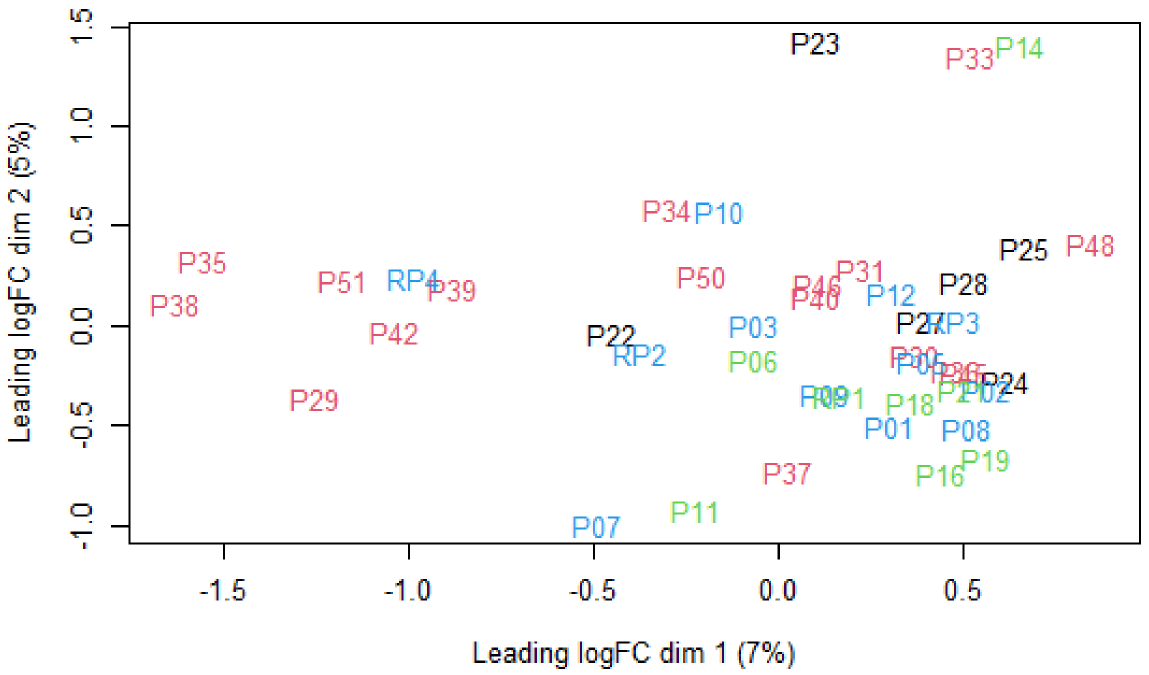

Additional File 1 Figure 12a. MDS Plot of blood samples (n=43) sequenced at MGI with standard chemistry reagents that underwent differential gene expression analysis: Group C (black, n=6), Group RT (red, n=17), Group RT-AS ≤ 2 (blue, n=12) and Group RT-AS ≥ 10 (green, n=8).

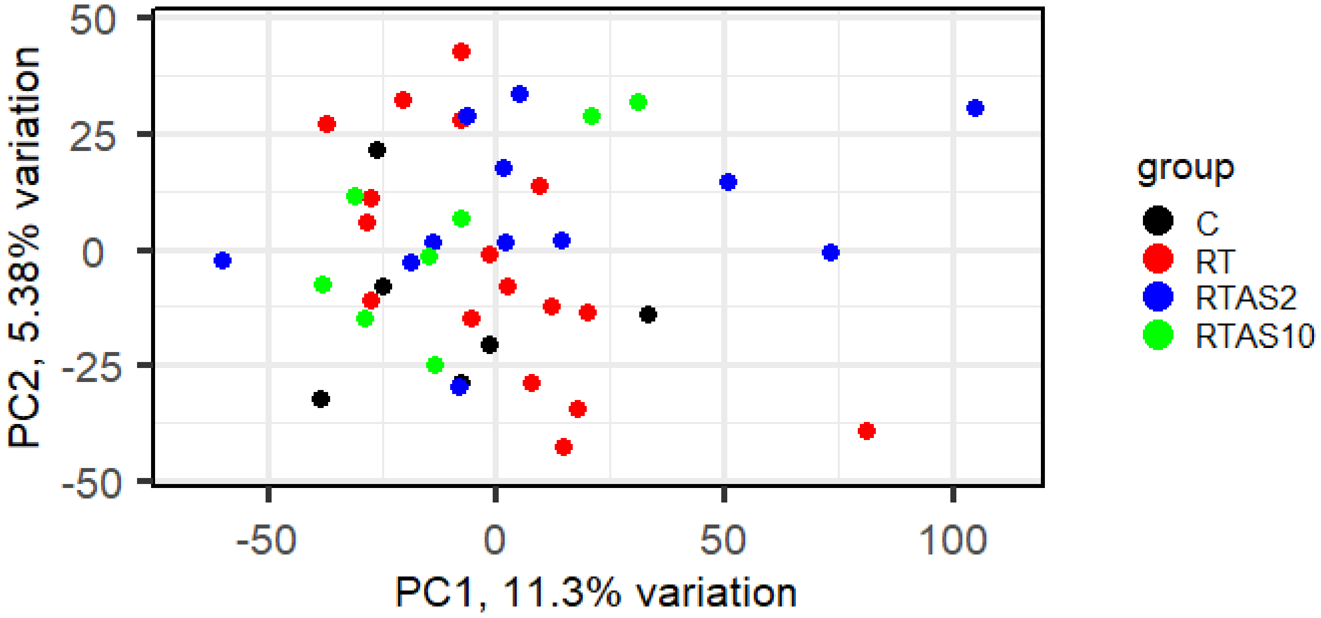

Additional File 1 Figure 12b. PCA Plot of blood samples (n=43) sequenced at MGI with standard chemistry reagents that underwent differential gene expression analysis: Group C (n=6), Group RT (n=17), Group RT-AS $\leq$ 2 (n=12) and Group RT-AS $\geq$ 10 (n=8).
