# Supplementary material for: An observational human study investigating the effect of anabolic androgenic steroid use on the transcriptome of skeletal muscle and whole blood using RNA-Seq
Source: BMC Med Genomics. 2023 May 3;16:94. doi: 10.1186/s12920-023-01512-z (PMC10157927; doi:10.1186/s12920-023-01512-z)
Supplement: Supplementary file 2 — Supplementary Material 2 [file 12920_2023_1512_MOESM2_ESM.pdf]

Additional File 2

**Sample**	**Group**	**Sequencing Location & Chemistry**	**Aligned Reads (Million)**	**Overall Alignment Rate %**
RP1_V1	RT-AS>10	UoB Standard	71.5	97.4
RP2_V1	RT-AS≤2	UoB Standard	72.7	97.3
RP2_V2	RP Visit 2	UoB Standard	40.1	96.6
RP3_V1	RT-AS≤2	UoB Standard	57.6	97.5
RP3_V2	RP Visit 2	UoB Standard	44.5	97.3
RP4_V1	RT-AS≤2	UoB Standard	86.9	97.1
RP4_V2	RP Visit 2	UoB Standard	56.5	97.3
RP5_V1	RT-AS≤2	UoB Standard	54.8	94.8
RP5_V2	RP Visit 2	UoB Standard	70.1	97.3
P01	RT-AS≤2	UoB Standard	42.6	96.3
P02	RT-AS≤2	UoB Standard	57.5	97.0
P03	RT-AS≤2	UoB Standard	67.6	97.3
P05	RT-AS≤2	UoB Standard	41.4	97.2
P07	RT-AS≤2	UoB Standard	46.9	97.2
P08	RT-AS≤2	UoB Standard	53.9	97.5
P09	RT-AS≤2	UoB Standard	56.9	95.0
P10	RT-AS≤2	UoB Standard	59.0	97.4
P12	RT-AS≤2	UoB Standard	110.6	97.7
P13	RT-AS≤2	UoB Standard	44.5	93.2
P06	RT-AS>10	UoB Standard	65.4	96.9
P11	RT-AS>10	UoB Standard	51.1	97.2
P14	RT-AS>10	UoB Standard	78.0	97.0
P16	RT-AS>10	UoB Standard	64.3	97.3
P17	RT-AS>10	UoB Standard	62.2	96.5
P18	RT-AS>10	UoB Standard	55.2	96.9
P19	RT-AS>10	UoB Standard	77.7	96.9
P20	RT-AS>10	UoB Standard	48.3	93.5
P21	RT-AS>10	UoB Standard	61.7	97.3
P22	C	UoB Standard	66.4	97.4
P23	C	UoB Standard	51.3	96.1
P24	C	UoB Standard	48.4	97.2
P25	C	UoB Standard	70.2	97.2
P26	C	UoB Standard	36.1	96.6
P27	C	UoB Standard	68.5	97.1
P28	C	UoB Standard	43.1	96.9
P29	RT	UoB Standard	88.0	97.2
P30	RT	UoB Standard	90.8	97.1
P31	RT	UoB Standard	58.2	96.6
P32	RT	UoB Standard	54.7	97.4
P33	RT	UoB Standard	45.2	96.9
P34	RT	UoB Standard	44.3	95.5
P35	RT	UoB Standard	33.2	94.7

P36	RT	UoB Standard	41.5	92.1
P37	RT	UoB Standard	55.1	95.8
P38	RT	UoB Standard	65.0	97.1
P39	RT	UoB Standard	51.0	95.2
P40	RT	UoB Standard	67.6	97.6
P42	RT	UoB Standard	90.5	97.6
P44	RT	UoB Standard	52.9	96.5
P45	RT	UoB Standard	42.5	96.9
P46	RT	UoB Standard	65.3	97.3
P48	RT	UoB Standard	58.8	96.9
P49	RT	UoB Standard	74.6	96.6
P50	RT	UoB Standard	56.9	97.3
P51	RT	UoB Standard	51.3	94.8
**Average ± SD**			59.5 ± 15.4	96.6 ± 1.2

Additional File 2 Table 1. HISAT2 alignment against the reference genome assembly (GRCh38.p5) with Ensemble 84 annotation utilising the publicly available grch38_tran pre-built HISAT index showing total aligned reads in million and overall alignment rate % for whole blood samples (n=55) sequenced at the University of Brighton (UoB) using standard reagents used in downstream DGE/GSEA analyses. RP: Returning Participant; _V1 or _V2: RP Visit 1 or 2; C: Control; RT: Resistance Trained; RT-AS≤2: RT Exposed to AAS ≤2 weeks before sampling; RT-AS>10: RT Exposed to AAS >10 weeks before sampling.

**Sample**	**Group**	**Sequencing Location & Chemistry**	**Aligned Reads (Million)**	**Overall Alignment Rate %**
RP1_V1	RT-AS>10	MGI Standard	61.8	97.1
RP2_V1	RT-AS≤2	MGI Standard	62.9	95.9
RP2_V2	RP Visit 2	MGI Standard	44.0	95.3
RP3_V1	RT-AS≤2	MGI Standard	63.9	97.3
RP3_V2	RP Visit 2	MGI Standard	49.9	95.1
RP4_V1	RT-AS≤2	MGI Standard	58.5	96.8
RP4_V2	RP Visit 2	MGI Standard	58.4	96.9
P01	RT-AS≤2	MGI Standard	47.7	95.9
P02	RT-AS≤2	MGI Standard	54.3	96.7
P03	RT-AS≤2	MGI Standard	58.2	97.0
P05	RT-AS≤2	MGI Standard	49.5	96.8
P07	RT-AS≤2	MGI Standard	60.0	95.9
P08	RT-AS≤2	MGI Standard	69.1	95.5
P09	RT-AS≤2	MGI Standard	55.2	91.4
P10	RT-AS≤2	MGI Standard	73.2	95.1
P12	RT-AS≤2	MGI Standard	86.5	95.5
P06	RT-AS>10	MGI Standard	54.2	96.6
P11	RT-AS>10	MGI Standard	50.9	95.7
P14	RT-AS>10	MGI Standard	87.9	96.7
P16	RT-AS>10	MGI Standard	59.2	95.6
P18	RT-AS>10	MGI Standard	74.4	95.3
P19	RT-AS>10	MGI Standard	51.7	96.5
P21	RT-AS>10	MGI Standard	60.3	95.5
P22	C	MGI Standard	68.3	97.2
P23	C	MGI Standard	55.8	95.9
P24	C	MGI Standard	47.6	95.9
P25	C	MGI Standard	63.5	96.7
P27	C	MGI Standard	56.0	95.9
P28	C	MGI Standard	45.6	96.8
P29	RT	MGI Standard	74.6	96.9
P30	RT	MGI Standard	77.9	96.8
P31	RT	MGI Standard	61.6	96.2
P33	RT	MGI Standard	51.0	95.5
P34	RT	MGI Standard	52.8	93.1
P35	RT	MGI Standard	41.0	91.6
P36	RT	MGI Standard	50.0	88.1
P37	RT	MGI Standard	59.1	93.0
P38	RT	MGI Standard	67.0	94.8
P39	RT	MGI Standard	50.5	92.3
P40	RT	MGI Standard	76.4	95.6
P42	RT	MGI Standard	69.4	95.7
P45	RT	MGI Standard	64.6	96.4
P46	RT	MGI Standard	51.3	97.1
P48	RT	MGI Standard	83.9	94.0
P50	RT	MGI Standard	47.6	95.1

P51	RT	MGI Standard	48.2	91.9
**Average ± SD**			59.9 ± 11.5	95.4 ± 1.9

Additional File 2 Table 2. HISAT2 alignment against the reference genome assembly (GRCh38.p5) with Ensemble 84 annotation utilising the publicly available grch38_tran pre-built HISAT index showing total aligned reads in million and overall alignment rate % for whole blood samples (n=46) sequenced by MGI, Latvia using standard reagents used in downstream DGE/GSEA analyses. RP: Returning Participant; _V1 or _V2: RP Visit 1 or 2; C: Control; RT: Resistance Trained; RT-AS≤2: RT Exposed to AAS ≤2 weeks before sampling; RT-AS>10: RT Exposed to AAS >10 weeks before sampling.

**Sample**	**Group**	**Sequencing Location & Chemistry**	**Aligned Reads (Million)**	**Overall Alignment Rate %**
RP1_V1	RT-AS>10	MGI Standard	50.6	97.0
RP2_V1	RT-AS≤2	MGI Standard	64.6	96.3
RP2_V2	RP Visit 2	MGI Standard	86.5	95.8
RP3_V1	RT-AS≤2	MGI Standard	55.1	96.9
RP3_V2	RP Visit 2	MGI Standard	71.0	93.4
RP4_V1	RT-AS≤2	MGI Standard	75.6	94.6
RP4_V2	RP Visit 2	MGI Standard	67.8	91.9
RP5_V1	RT-AS≤2	MGI Standard	84.9	94.2
P01	RT-AS≤2	MGI Standard	83.8	94.2
P02	RT-AS≤2	MGI Standard	71.8	94.6
P03	RT-AS≤2	MGI Standard	64.1	94.1
P04	RT-AS≤2	MGI Standard	80.2	96.5
P05	RT-AS≤2	MGI Standard	80.0	94.6
P07	RT-AS≤2	MGI Standard	59.7	97.3
P08	RT-AS≤2	MGI Standard	71.3	93.7
P09	RT-AS≤2	MGI Standard	78.2	93.9
P10	RT-AS≤2	MGI Standard	57.2	93.7
P12	RT-AS≤2	MGI Standard	56.2	97.1
P13	RT-AS≤2	MGI Standard	113.7	96.3
P06	RT-AS>10	MGI Standard	60.8	97.3
P11	RT-AS>10	MGI Standard	70.8	96.7
P14	RT-AS>10	MGI Standard	74.8	94.5
P15	RT-AS>10	MGI Standard	77.7	93.3
P16	RT-AS>10	MGI Standard	83.2	93.2
P17	RT-AS>10	MGI Standard	66.1	96.8
P18	RT-AS>10	MGI Standard	71.5	96.8
P19	RT-AS>10	MGI Standard	57.9	97.0
P20	RT-AS>10	MGI Standard	71.8	96.3
P21	RT-AS>10	MGI Standard	67.9	97.0
P23	C	MGI Standard	70.6	96.9
P24	C	MGI Standard	72.0	95.6
P26	C	MGI Standard	68.2	92.1
P27	C	MGI Standard	56.0	96.6
P28	C	MGI Standard	40.6	97.5
P30	RT	MGI Standard	57.1	95.3
P31	RT	MGI Standard	55.4	97.4
P32	RT	MGI Standard	56.2	96.9
P33	RT	MGI Standard	65.6	96.6
P34	RT	MGI Standard	73.0	95.4
P35	RT	MGI Standard	56.4	97.5
P36	RT	MGI Standard	71.1	93.3
P37	RT	MGI Standard	97.4	91.8
P41	RT	MGI Standard	79.4	93.8
P42	RT	MGI Standard	60.8	97.4
P43	RT	MGI Standard	73.8	94.2

P45	RT	MGI Standard	71.1	96.8
P46	RT	MGI Standard	70.1	94.5
P48	RT	MGI Standard	59.3	93.6
P49	RT	MGI Standard	70.9	93.7
P50	RT	MGI Standard	67.0	96.0
P51	RT	MGI Standard	66.8	94.3
**Average ± SD**			69.3 ± 12.2	95.3 ± 1.7

Additional File 2 Table 3. HISAT2 alignment against the reference genome assembly (GRCh38.p5) with Ensemble 84 annotation utilising the publicly available grch38_tran pre-built HISAT index showing total aligned reads in million and overall alignment rate % for muscle samples (n=51) sequenced by MGI, Latvia using standard reagents used in downstream DGE/GSEA analyses. RP: Returning Participant; _V1 or _V2: RP Visit 1 or 2; C: Control; RT: Resistance Trained; RT-AS≤2: RT Exposed to AAS ≤2 weeks before sampling; RT-AS>10: RT Exposed to AAS >10 weeks before sampling.

**Sample**	**Group**	**Sequencing Location & Chemistry**	**Aligned Reads (Million)**	**Overall Alignment Rate %**
RP1_V1	RT-AS>10	MGI CoolMPS	54.8	98.2
RP2_V1	RT-AS≤2	MGI CoolMPS	64.6	98.8
RP2_V2	RP Visit 2	MGI CoolMPS	71.9	98.8
RP3_V1	RT-AS≤2	MGI CoolMPS	60.5	98.3
RP3_V2	RP Visit 2	MGI CoolMPS	78.2	97.9
RP4_V1	RT-AS≤2	MGI CoolMPS	84.9	98.8
RP4_V2	RP Visit 2	MGI CoolMPS	58.8	96.8
RP5_V1	RT-AS≤2	MGI CoolMPS	80.1	98.7
P01	RT-AS≤2	MGI CoolMPS	70.2	98.3
P02	RT-AS≤2	MGI CoolMPS	73.8	98.9
P03	RT-AS≤2	MGI CoolMPS	75.0	98.4
P04	RT-AS≤2	MGI CoolMPS	90.2	98.3
P05	RT-AS≤2	MGI CoolMPS	82.9	98.8
P07	RT-AS≤2	MGI CoolMPS	62.5	98.8
P08	RT-AS≤2	MGI CoolMPS	73.7	98.5
P09	RT-AS≤2	MGI CoolMPS	77.2	98.7
P10	RT-AS≤2	MGI CoolMPS	60.3	98.8
P12	RT-AS≤2	MGI CoolMPS	57.8	98.6
P13	RT-AS≤2	MGI CoolMPS	72.1	98.9
P06	RT-AS>10	MGI CoolMPS	64.9	98.3
P11	RT-AS>10	MGI CoolMPS	79.2	98.3
P14	RT-AS>10	MGI CoolMPS	79.2	98.0
P15	RT-AS>10	MGI CoolMPS	79.2	97.8
P16	RT-AS>10	MGI CoolMPS	86.6	98.7
P17	RT-AS>10	MGI CoolMPS	73.0	98.4
P18	RT-AS>10	MGI CoolMPS	79.2	98.5
P19	RT-AS>10	MGI CoolMPS	59.5	98.7
P20	RT-AS>10	MGI CoolMPS	72.0	98.7
P21	RT-AS>10	MGI CoolMPS	60.3	98.6
P23	C	MGI CoolMPS	65.4	98.7
P24	C	MGI CoolMPS	68.9	98.3
P26	C	MGI CoolMPS	83.7	98.1
P27	C	MGI CoolMPS	58.7	97.8
P28	C	MGI CoolMPS	55.7	98.8
P30	RT	MGI CoolMPS	45.6	98.5
P31	RT	MGI CoolMPS	59.6	98.3
P32	RT	MGI CoolMPS	60.6	98.7
P33	RT	MGI CoolMPS	73.4	98.3
P34	RT	MGI CoolMPS	70.1	98.6
P35	RT	MGI CoolMPS	61.7	98.6
P36	RT	MGI CoolMPS	73.2	97.9
P37	RT	MGI CoolMPS	100.5	98.2
P41	RT	MGI CoolMPS	82.4	98.6
P42	RT	MGI CoolMPS	66.3	98.6

P43	RT	MGI CoolMPS	89.5	98.4
P45	RT	MGI CoolMPS	78.7	98.4
P46	RT	MGI CoolMPS	77.5	98.5
P48	RT	MGI CoolMPS	66.3	97.5
P49	RT	MGI CoolMPS	74.3	98.7
P50	RT	MGI CoolMPS	66.2	98.9
P51	RT	MGI CoolMPS	64.9	98.6
**Average ± SD**			71.1 ± 10.7	98.4 ± 0.4

Additional File 2 Table 4. HISAT2 alignment against the reference genome assembly (GRCh38.p5) with Ensemble 84 annotation utilising the publicly available grch38_tran pre-built HISAT index showing total aligned reads in million and overall alignment rate % for muscle samples (n=51) sequenced by MGI, Latvia using CoolMPS reagents used in downstream DGE/GSEA analyses. RP: Returning Participant; _V1 or _V2: RP Visit 1 or 2; C: Control; RT: Resistance Trained; RT-AS≤2: RT Exposed to AAS ≤2 weeks before sampling; RT-AS>10: RT Exposed to AAS >10 weeks before sampling.

**Sample**	**Group**	**Sequencing Location & Chemistry**	**Total Reads (Million)**	**% CDS Exons**	**% Introns**
RP1_V1	RT-AS>10	UoB Standard	139.4	46.6	31.2
RP2_V1	RT-AS≤2	UoB Standard	141.4	47.9	29.2
RP2_V2	RP Visit 2	UoB Standard	77.3	54.3	24.4
RP3_V1	RT-AS≤2	UoB Standard	112.2	53.5	25.9
RP3_V2	RP Visit 2	UoB Standard	86.6	56.5	22.1
RP4_V1	RT-AS≤2	UoB Standard	168.7	53.1	25.7
RP4_V2	RP Visit 2	UoB Standard	110.0	48.6	28.9
RP5_V1	RT-AS≤2	UoB Standard	103.9	46.2	28.8
RP5_V2	RP Visit 2	UoB Standard	136.5	46.5	30.6
P01	RT-AS≤2	UoB Standard	82.0	48.1	29.4
P02	RT-AS≤2	UoB Standard	111.5	50.6	26.7
P03	RT-AS≤2	UoB Standard	131.6	43.0	33.6
P05	RT-AS≤2	UoB Standard	80.5	63.6	18.2
P07	RT-AS≤2	UoB Standard	91.2	62.8	17.1
P08	RT-AS≤2	UoB Standard	105.1	50.2	27.3
P09	RT-AS≤2	UoB Standard	108.2	49.8	21.2
P10	RT-AS≤2	UoB Standard	115.0	50.3	27.7
P12	RT-AS≤2	UoB Standard	216.1	61.0	17.6
P13	RT-AS≤2	UoB Standard	83.0	32.5	26.4
P06	RT-AS>10	UoB Standard	126.8	51.4	27.1
P11	RT-AS>10	UoB Standard	99.3	51.2	26.1
P14	RT-AS>10	UoB Standard	151.3	55.4	23.2
P16	RT-AS>10	UoB Standard	125.2	50.3	26.8
P17	RT-AS>10	UoB Standard	120.1	55.0	24.7
P18	RT-AS>10	UoB Standard	106.9	45.9	30.8
P19	RT-AS>10	UoB Standard	150.6	53.6	24.9
P20	RT-AS>10	UoB Standard	90.3	42.1	26.9
P21	RT-AS>10	UoB Standard	120.0	46.3	30.0
P22	C	UoB Standard	129.4	44.8	32.1
P23	C	UoB Standard	98.6	48.3	28.2
P24	C	UoB Standard	94.1	54.5	26.1
P25	C	UoB Standard	136.5	54.8	25.1
P26	C	UoB Standard	69.7	48.2	29.9
P27	C	UoB Standard	133.1	50.9	26.8
P28	C	UoB Standard	83.6	49.9	28.6
P29	RT	UoB Standard	171.1	45.0	32.7
P30	RT	UoB Standard	176.4	46.8	31.6
P31	RT	UoB Standard	112.4	61.7	17.8
P32	RT	UoB Standard	106.5	49.4	27.3
P33	RT	UoB Standard	87.6	69.2	12.9
P34	RT	UoB Standard	84.7	51.7	21.8
P35	RT	UoB Standard	62.9	53.9	20.9
P36	RT	UoB Standard	76.5	37.2	25.3
P37	RT	UoB Standard	105.6	40.9	31.3
P38	RT	UoB Standard	126.3	42.5	34.0

P39	RT	UoB Standard	97.1	43.5	27.9
P40	RT	UoB Standard	132.0	53.4	25.0
P42	RT	UoB Standard	176.6	53.5	23.8
P44	RT	UoB Standard	102.0	48.5	28.0
P45	RT	UoB Standard	82.3	58.3	21.7
P46	RT	UoB Standard	126.9	43.7	33.6
P48	RT	UoB Standard	113.9	50.6	22.8
P49	RT	UoB Standard	144.2	49.7	27.9
P50	RT	UoB Standard	110.7	50.3	28.3
P51	RT	UoB Standard	97.3	47.4	25.4
**Average ± SD**			115.1 ± 30.2	50.3 ± 6.4	26.4 ± 4.5

Additional File 2 Table 5. RSeQC read distribution for whole blood samples (n=55) sequenced at the University of Brighton (UoB) using standard reagents used in downstream DGE/GSEA analyses including the total reads, % CDS Exons tag counts and % Intron tag counts aligned to the reference genome assembly (GRCh38.p5) with Ensemble 84 annotation as the publicly available HISAT2 index was utilised. RP: Returning Participant; _V1 or _V2: RP Visit 1 or 2; C: Control; RT: Resistance Trained; RT-AS≤2: RT Exposed to AAS ≤2 weeks before sampling; RT-AS>10: RT Exposed to AAS >10 weeks before sampling; CDS: coding sequences.

Sample	Group	Sequencing Location & Chemistry	Total Reads (Million)	% CDS Exons	% Introns
RP1_V1	RT-AS>10	MGI Standard	120.0	46.4	31.3
RP2_V1	RT-AS≤2	MGI Standard	120.7	47.7	29.2
RP2_V2	RP Visit 2	MGI Standard	83.9	53.5	24.7
RP3_V1	RT-AS≤2	MGI Standard	124.2	53.6	25.7
RP3_V2	RP Visit 2	MGI Standard	95.0	56.3	22.4
RP4_V1	RT-AS≤2	MGI Standard	113.3	52.4	26.3
RP4_V2	RP Visit 2	MGI Standard	113.1	47.9	29.2
P01	RT-AS≤2	MGI Standard	91.5	48.0	29.5
P02	RT-AS≤2	MGI Standard	105.0	50.3	26.8
P03	RT-AS≤2	MGI Standard	112.8	43.0	33.6
P05	RT-AS≤2	MGI Standard	95.8	63.0	18.5
P07	RT-AS≤2	MGI Standard	115.1	61.3	17.4
P08	RT-AS≤2	MGI Standard	132.0	49.8	27.5
P09	RT-AS≤2	MGI Standard	101.0	49.4	21.3
P10	RT-AS≤2	MGI Standard	139.2	49.0	28.5
P12	RT-AS≤2	MGI Standard	165.2	60.9	17.5
P06	RT-AS>10	MGI Standard	104.8	50.9	27.5
P11	RT-AS>10	MGI Standard	97.5	50.5	26.3
P14	RT-AS>10	MGI Standard	170.0	55.3	23.3
P16	RT-AS>10	MGI Standard	113.1	50.4	26.7
P18	RT-AS>10	MGI Standard	141.8	44.8	31.2
P19	RT-AS>10	MGI Standard	99.8	52.3	25.9
P21	RT-AS>10	MGI Standard	115.1	46.7	29.6
P22	C	MGI Standard	132.8	44.7	32.2
P23	C	MGI Standard	106.9	47.1	29.3
P24	C	MGI Standard	91.2	53.4	26.6
P25	C	MGI Standard	122.9	54.7	25.3
P27	C	MGI Standard	107.4	50.3	27.1
P28	C	MGI Standard	88.2	49.8	28.5
P29	RT	MGI Standard	144.7	45.3	32.6
P30	RT	MGI Standard	150.9	46.8	31.8
P31	RT	MGI Standard	118.5	61.1	18.1
P33	RT	MGI Standard	97.5	67.4	13.5
P34	RT	MGI Standard	98.4	51.3	21.8
P35	RT	MGI Standard	75.2	53.5	21.0
P36	RT	MGI Standard	88.1	37.3	25.3
P37	RT	MGI Standard	110.0	41.0	31.0
P38	RT	MGI Standard	127.2	43.1	33.5
P39	RT	MGI Standard	93.2	43.8	27.7
P40	RT	MGI Standard	146.0	52.5	25.3
P42	RT	MGI Standard	132.9	52.6	24.1
P45	RT	MGI Standard	124.6	57.9	21.8
P46	RT	MGI Standard	99.6	43.2	33.8
P48	RT	MGI Standard	157.8	50.1	23.1
P50	RT	MGI Standard	90.5	50.4	28.2

P51	RT	MGI Standard	88.6	47.4	25.6
**Average ± SD**			114.4 ± 22.5	50.6 ± 6.0	26.2 ± 4.7

Additional File 2 Table 6. RSeQC read distribution for whole blood samples (n=46) sequenced at MGI, Latvia using standard reagents used in downstream DGE/GSEA analyses including the total reads, % CDS Exons tag counts and % Intron tag counts aligned to the reference genome assembly (GRCh38.p5) with Ensemble 84 annotation as the publicly available HISAT2 index was utilised. RP: Returning Participant; _V1 or _V2: RP Visit 1 or 2; C: Control; RT: Resistance Trained; RT-AS≤2: RT Exposed to AAS ≤2 weeks before sampling; RT-AS>10: RT Exposed to AAS >10 weeks before sampling; CDS: coding sequences.

**Sample**	**Group**	**Sequencing Location & Chemistry**	**Total Reads (Millions)**	**% CDS Exons**	**% Introns**
RP1_V1	RT-AS>10	MGI Standard	98.1	70.6	13.6
RP2_V1	RT-AS≤2	MGI Standard	124.5	68.8	15.9
RP2_V2	RP Visit 2	MGI Standard	165.6	66.4	17.9
RP3_V1	RT-AS≤2	MGI Standard	106.8	64.8	17.9
RP3_V2	RP Visit 2	MGI Standard	132.5	64.6	18.4
RP4_V1	RT-AS≤2	MGI Standard	142.9	67.3	17.2
RP4_V2	RP Visit 2	MGI Standard	124.5	60.6	20.7
RP5_V1	RT-AS≤2	MGI Standard	159.9	65.3	18.6
P01	RT-AS≤2	MGI Standard	158.0	66.9	17.2
P02	RT-AS≤2	MGI Standard	135.8	66.8	17.7
P03	RT-AS≤2	MGI Standard	120.6	67.4	17.1
P04	RT-AS≤2	MGI Standard	154.9	67.6	16.4
P05	RT-AS≤2	MGI Standard	151.3	65.9	17.8
P07	RT-AS≤2	MGI Standard	116.2	65.6	18.7
P08	RT-AS≤2	MGI Standard	133.6	67.8	16.5
P09	RT-AS≤2	MGI Standard	146.9	66.1	17.4
P10	RT-AS≤2	MGI Standard	107.1	66.5	17.4
P12	RT-AS≤2	MGI Standard	109.2	65.6	18.3
P13	RT-AS≤2	MGI Standard	219.0	65.4	18.9
P06	RT-AS>10	MGI Standard	118.3	66.7	17.8
P11	RT-AS>10	MGI Standard	136.8	65.5	18.7
P14	RT-AS>10	MGI Standard	141.3	66.1	18.2
P15	RT-AS>10	MGI Standard	145.1	60.2	19.9
P16	RT-AS>10	MGI Standard	155.1	66.9	17.0
P17	RT-AS>10	MGI Standard	128.0	68.0	16.2
P18	RT-AS>10	MGI Standard	138.5	67.5	16.9
P19	RT-AS>10	MGI Standard	112.3	66.5	17.8
P20	RT-AS>10	MGI Standard	138.3	65.0	19.8
P21	RT-AS>10	MGI Standard	131.8	64.9	19.4
P23	C	MGI Standard	136.9	61.8	21.9
P24	C	MGI Standard	137.5	63.6	20.6
P26	C	MGI Standard	125.6	69.2	13.9
P27	C	MGI Standard	108.3	70.4	11.9
P28	C	MGI Standard	79.1	63.8	20.5
P30	RT	MGI Standard	108.8	74.8	8.2
P31	RT	MGI Standard	107.9	66.8	17.5
P32	RT	MGI Standard	109.0	65.9	18.4
P33	RT	MGI Standard	126.8	68.0	15.6
P34	RT	MGI Standard	139.3	66.7	18.0
P35	RT	MGI Standard	110.1	71.0	13.8
P36	RT	MGI Standard	132.6	69.2	14.7
P37	RT	MGI Standard	178.9	64.6	18.5
P41	RT	MGI Standard	148.9	63.2	20.9
P42	RT	MGI Standard	118.5	67.7	17.8
P43	RT	MGI Standard	139.0	68.8	15.1

P45	RT	MGI Standard	137.6	73.4	10.9
P46	RT	MGI Standard	132.4	65.9	17.6
P48	RT	MGI Standard	111.0	72.8	10.6
P49	RT	MGI Standard	132.9	65.4	18.7
P50	RT	MGI Standard	128.6	66.9	17.7
P51	RT	MGI Standard	125.9	67.7	16.4
**Average ± SD**			131.9 ± 22.5	66.8 ± 2.8	17.1 ± 2.7

Additional File 2 Table 7. RSeQC read distribution for muscle samples (N=51) sequenced by MGI, Latvia using standard reagents used in downstream DGE/GSEA analyses including the total reads, % CDS Exons tag counts and % Intron tag counts aligned to the reference genome assembly (GRCh38.p5) with Ensemble 84 annotation as the publicly available HISAT2 index was utilised. RP: Returning Participant; _V1 or _V2: RP Visit 1 or 2; C: Control; RT: Resistance Trained; RT-AS≤2: RT Exposed to AAS ≤2 weeks before sampling; RT-AS>10: RT Exposed to AAS >10 weeks before sampling; CDS: coding sequences.

Sample	Group	Sequencing Location & Chemistry	Total Reads (Millions)	% CDS Exons	% Introns
RP1_V1	RT-AS>10	MGI CoolMPS	107.7	70.6	13.6
RP2_V1	RT-AS≤2	MGI CoolMPS	127.6	68.6	16.1
RP2_V2	RP Visit 2	MGI CoolMPS	142.1	66.4	18.1
RP3_V1	RT-AS≤2	MGI CoolMPS	118.9	64.8	18.0
RP3_V2	RP Visit 2	MGI CoolMPS	153.3	64.4	18.4
RP4_V1	RT-AS≤2	MGI CoolMPS	167.7	67.3	17.3
RP4_V2	RP Visit 2	MGI CoolMPS	113.9	59.5	20.5
RP5_V1	RT-AS≤2	MGI CoolMPS	158.1	65.6	18.6
P01	RT-AS≤2	MGI CoolMPS	138.1	66.9	17.1
P02	RT-AS≤2	MGI CoolMPS	146.0	67.1	17.6
P03	RT-AS≤2	MGI CoolMPS	147.6	67.3	17.0
P04	RT-AS≤2	MGI CoolMPS	177.2	67.1	16.7
P05	RT-AS≤2	MGI CoolMPS	163.8	66.3	17.6
P07	RT-AS≤2	MGI CoolMPS	123.5	65.8	18.6
P08	RT-AS≤2	MGI CoolMPS	145.2	67.5	16.6
P09	RT-AS≤2	MGI CoolMPS	152.4	66.2	17.3
P10	RT-AS≤2	MGI CoolMPS	119.0	66.3	17.4
P12	RT-AS≤2	MGI CoolMPS	114.0	66.0	18.1
P13	RT-AS≤2	MGI CoolMPS	142.7	65.4	19.0
P06	RT-AS>10	MGI CoolMPS	127.6	66.7	17.9
P11	RT-AS>10	MGI CoolMPS	155.8	64.8	19.1
P14	RT-AS>10	MGI CoolMPS	155.3	65.7	18.1
P15	RT-AS>10	MGI CoolMPS	155.0	59.4	20.0
P16	RT-AS>10	MGI CoolMPS	170.9	66.6	17.1
P17	RT-AS>10	MGI CoolMPS	143.6	67.6	16.4
P18	RT-AS>10	MGI CoolMPS	155.9	67.0	17.2
P19	RT-AS>10	MGI CoolMPS	117.4	66.9	17.6
P20	RT-AS>10	MGI CoolMPS	142.1	65.1	19.9
P21	RT-AS>10	MGI CoolMPS	118.9	65.2	19.2
P23	C	MGI CoolMPS	129.0	62.5	21.5
P24	C	MGI CoolMPS	135.4	63.7	20.4
P26	C	MGI CoolMPS	164.2	68.4	13.9
P27	C	MGI CoolMPS	114.8	70.8	11.9
P28	C	MGI CoolMPS	110.1	64.0	20.3
P30	RT	MGI CoolMPS	89.8	74.6	8.2
P31	RT	MGI CoolMPS	117.2	66.8	17.6
P32	RT	MGI CoolMPS	119.6	66.2	18.2
P33	RT	MGI CoolMPS	144.4	67.4	16.0
P34	RT	MGI CoolMPS	138.1	66.6	18.0
P35	RT	MGI CoolMPS	121.7	70.9	13.9
P36	RT	MGI CoolMPS	143.2	69.1	14.6
P37	RT	MGI CoolMPS	197.4	64.0	18.4
P41	RT	MGI CoolMPS	162.5	63.0	21.0
P42	RT	MGI CoolMPS	130.7	67.8	17.8
P43	RT	MGI CoolMPS	176.1	68.6	15.0

P45	RT	MGI CoolMPS	154.9	73.2	11.0
P46	RT	MGI CoolMPS	152.7	65.2	17.8
P48	RT	MGI CoolMPS	129.4	72.3	10.6
P49	RT	MGI CoolMPS	146.7	65.1	18.8
P50	RT	MGI CoolMPS	130.9	66.7	17.8
P51	RT	MGI CoolMPS	128.1	67.8	16.3
**Average ± SD**			140.0 ± 21.1	66.6 ± 2.8	17.2 ± 2.6

Additional File 2 Table 8. RSeQC read distribution for muscle samples (N=51) sequenced by MGI, Latvia using CoolMPS reagents used in downstream DGE/GSEA analyses including the total reads, % CDS Exons tag counts and % Intron tag counts aligned to the reference genome assembly (GRCh38.p5) with Ensemble 84 annotation as the publicly available HISAT2 index was utilised. RP: Returning Participant; _V1 or _V2: RP Visit 1 or 2; C: Control; RT: Resistance Trained; RT-AS≤2: RT Exposed to AAS ≤2 weeks before sampling; RT-AS>10: RT Exposed to AAS >10 weeks before sampling; CDS: coding sequences.

Sample	Group	Sequencing Location & Chemistry	Processed Reads (Million)	Mapped Reads %
RP1_V1	RT-AS>10	UoB Standard	71.5	51.8
RP2_V1	RT-AS≤2	UoB Standard	72.7	53.8
RP2_V2	RP Visit 2	UoB Standard	40.1	59.6
RP3_V1	RT-AS≤2	UoB Standard	57.6	58.3
RP3_V2	RP Visit 2	UoB Standard	44.5	63.1
RP4_V1	RT-AS≤2	UoB Standard	86.9	57.7
RP4_V2	RP Visit 2	UoB Standard	56.5	55.3
RP5_V1	RT-AS≤2	UoB Standard	54.8	51.2
RP5_V2	RP Visit 2	UoB Standard	70.1	53.2
P01	RT-AS≤2	UoB Standard	42.6	53.6
P02	RT-AS≤2	UoB Standard	57.5	55.4
P03	RT-AS≤2	UoB Standard	67.6	50.1
P05	RT-AS≤2	UoB Standard	41.4	66.6
P07	RT-AS≤2	UoB Standard	46.9	67.9
P08	RT-AS≤2	UoB Standard	53.9	56.2
P09	RT-AS≤2	UoB Standard	56.9	51.2
P10	RT-AS≤2	UoB Standard	59.0	55.6
P12	RT-AS≤2	UoB Standard	110.6	68.3
P13	RT-AS≤2	UoB Standard	44.5	33.5
P06	RT-AS>10	UoB Standard	65.4	56.4
P11	RT-AS>10	UoB Standard	51.1	58.2
P14	RT-AS>10	UoB Standard	78.0	61.7
P16	RT-AS>10	UoB Standard	64.3	56.5
P17	RT-AS>10	UoB Standard	62.2	58.5
P18	RT-AS>10	UoB Standard	55.2	51.5
P19	RT-AS>10	UoB Standard	77.7	58.2
P20	RT-AS>10	UoB Standard	48.3	45.8
P21	RT-AS>10	UoB Standard	61.7	53.2
P22	C	UoB Standard	66.4	50.9
P23	C	UoB Standard	51.3	54.1
P24	C	UoB Standard	48.4	57.4
P25	C	UoB Standard	70.2	58.9
P26	C	UoB Standard	36.1	53.3
P27	C	UoB Standard	68.5	56.7
P28	C	UoB Standard	43.1	54.5
P29	RT	UoB Standard	88.0	50.7
P30	RT	UoB Standard	90.8	51.8
P31	RT	UoB Standard	58.2	67.5
P32	RT	UoB Standard	54.7	57.1
P33	RT	UoB Standard	45.2	74.8
P34	RT	UoB Standard	44.3	54.7
P35	RT	UoB Standard	33.2	57.6
P36	RT	UoB Standard	41.5	37.7
P37	RT	UoB Standard	55.1	45.2
P38	RT	UoB Standard	65.0	48.8

P39	RT	UoB Standard	51.0	48.5
P40	RT	UoB Standard	67.6	59.3
P42	RT	UoB Standard	90.5	59.8
P44	RT	UoB Standard	52.9	55.4
P45	RT	UoB Standard	42.5	62.7
P46	RT	UoB Standard	65.3	49.5
P48	RT	UoB Standard	58.8	57.6
P49	RT	UoB Standard	74.6	55.6
P50	RT	UoB Standard	56.9	55.1
P51	RT	UoB Standard	51.3	51.5
**Average ± SD**			59.5 ± 15.4	55.4 ± 6.9

Additional File 2 Table 9. Number of processed reads and percentage of mapped reads by Salmon for whole blood samples (n=55) sequenced at the University of Brighton (UoB) using standard reagents used in downstream DGE/GSEA analyses. Salmon indexing against the publicly available hg38 full decoy-aware salmon index. RP: Returning Participant; _V1 or _V2: RP Visit 1 or 2; C: Control; RT: Resistance Trained; RT-AS≤2: RT Exposed to AAS ≤2 weeks before sampling; RT-AS>10: RT Exposed to AAS >10 weeks before sampling.

**Sample**	**Group**	**Sequencing Location & Chemistry**	**Processed Reads (Million)**	**Mapped Reads %**
RP1_V1	RT-AS>10	MGI Standard	61.8	51.7
RP2_V1	RT-AS≤2	MGI Standard	62.9	53.5
RP2_V2	RP Visit 2	MGI Standard	44.0	58.8
RP3_V1	RT-AS≤2	MGI Standard	63.9	58.4
RP3_V2	RP Visit 2	MGI Standard	49.9	62.2
RP4_V1	RT-AS≤2	MGI Standard	58.5	57.0
RP4_V2	RP Visit 2	MGI Standard	58.4	55.0
P01	RT-AS≤2	MGI Standard	47.7	53.4
P02	RT-AS≤2	MGI Standard	54.3	55.1
P03	RT-AS≤2	MGI Standard	58.2	50.0
P05	RT-AS≤2	MGI Standard	49.5	66.1
P07	RT-AS≤2	MGI Standard	60.0	66.9
P08	RT-AS≤2	MGI Standard	69.1	55.3
P09	RT-AS≤2	MGI Standard	55.2	49.9
P10	RT-AS≤2	MGI Standard	73.2	54.3
P12	RT-AS≤2	MGI Standard	86.5	68.0
P06	RT-AS>10	MGI Standard	54.2	55.8
P11	RT-AS>10	MGI Standard	50.9	57.6
P14	RT-AS>10	MGI Standard	87.9	61.6
P16	RT-AS>10	MGI Standard	59.2	56.3
P18	RT-AS>10	MGI Standard	74.4	50.6
P19	RT-AS>10	MGI Standard	51.7	57.0
P21	RT-AS>10	MGI Standard	60.3	53.2
P22	C	MGI Standard	68.3	50.8
P23	C	MGI Standard	55.8	53.0
P24	C	MGI Standard	47.6	56.4
P25	C	MGI Standard	63.5	58.6
P27	C	MGI Standard	56.0	56.1
P28	C	MGI Standard	45.6	54.5
P29	RT	MGI Standard	74.6	50.5
P30	RT	MGI Standard	77.9	51.7
P31	RT	MGI Standard	61.6	66.8
P33	RT	MGI Standard	51.0	73.5
P34	RT	MGI Standard	52.8	53.8
P35	RT	MGI Standard	41.0	56.6
P36	RT	MGI Standard	50.0	36.9
P37	RT	MGI Standard	59.1	44.6
P38	RT	MGI Standard	67.0	48.9
P39	RT	MGI Standard	50.5	48.1
P40	RT	MGI Standard	76.4	58.5
P42	RT	MGI Standard	69.4	59.1
P45	RT	MGI Standard	64.6	62.1
P46	RT	MGI Standard	51.3	49.3
P48	RT	MGI Standard	83.9	56.6
P50	RT	MGI Standard	47.6	54.8

P51	RT	MGI Standard	48.2	50.8
**Average ± SD**			59.9 ± 11.5	55.6 ± 6.4

Additional File 2 Table 10. Number of processed reads and percentage of mapped reads by Salmon for whole blood samples (n=46) sequenced at MGI, Latvia using standard reagents used in downstream DGE/GSEA analyses. Salmon indexing against the publicly available hg38 full decoy-aware salmon index. RP: Returning Participant; _V1 or _V2: RP Visit 1 or 2; C: Control; RT: Resistance Trained; RT-AS≤2: RT Exposed to AAS ≤2 weeks before sampling; RT-AS>10: RT Exposed to AAS >10 weeks before sampling.

Sample	Group	Sequencing Location & Chemistry	Processed Reads (Million)	Mapped Reads %
RP1_V1	RT-AS>10	MGI Standard	50.6	75.7
RP2_V1	RT-AS≤2	MGI Standard	64.6	72.7
RP2_V2	RP Visit 2	MGI Standard	86.5	70.0
RP3_V1	RT-AS≤2	MGI Standard	55.1	68.0
RP3_V2	RP Visit 2	MGI Standard	71.0	68.3
RP4_V1	RT-AS≤2	MGI Standard	75.6	70.2
RP4_V2	RP Visit 2	MGI Standard	67.8	60.5
RP5_V1	RT-AS≤2	MGI Standard	84.9	68.7
P01	RT-AS≤2	MGI Standard	83.8	70.2
P02	RT-AS≤2	MGI Standard	71.8	70.1
P03	RT-AS≤2	MGI Standard	64.1	70.6
P04	RT-AS≤2	MGI Standard	80.2	71.7
P05	RT-AS≤2	MGI Standard	80.0	69.8
P07	RT-AS≤2	MGI Standard	59.7	69.3
P08	RT-AS≤2	MGI Standard	71.3	70.8
P09	RT-AS≤2	MGI Standard	78.2	69.1
P10	RT-AS≤2	MGI Standard	57.2	69.6
P12	RT-AS≤2	MGI Standard	56.2	69.6
P13	RT-AS≤2	MGI Standard	113.7	68.6
P06	RT-AS>10	MGI Standard	60.8	70.2
P11	RT-AS>10	MGI Standard	70.8	68.9
P14	RT-AS>10	MGI Standard	74.8	67.8
P15	RT-AS>10	MGI Standard	77.7	60.6
P16	RT-AS>10	MGI Standard	83.2	69.7
P17	RT-AS>10	MGI Standard	66.1	72.5
P18	RT-AS>10	MGI Standard	71.5	71.7
P19	RT-AS>10	MGI Standard	57.9	70.6
P20	RT-AS>10	MGI Standard	71.8	67.9
P21	RT-AS>10	MGI Standard	67.9	68.6
P23	C	MGI Standard	70.6	65.1
P24	C	MGI Standard	72.0	66.2
P26	C	MGI Standard	68.2	71.1
P27	C	MGI Standard	56.0	75.4
P28	C	MGI Standard	40.6	67.5
P30	RT	MGI Standard	57.1	80.5
P31	RT	MGI Standard	55.4	70.3
P32	RT	MGI Standard	56.2	69.7
P33	RT	MGI Standard	65.6	72.7
P34	RT	MGI Standard	73.0	69.4
P35	RT	MGI Standard	56.4	75.8
P36	RT	MGI Standard	71.1	73.2
P37	RT	MGI Standard	97.4	65.7
P41	RT	MGI Standard	79.4	65.4
P42	RT	MGI Standard	60.8	71.1
P43	RT	MGI Standard	73.8	73.0

P45	RT	MGI Standard	71.1	79.9
P46	RT	MGI Standard	70.1	67.4
P48	RT	MGI Standard	59.3	77.5
P49	RT	MGI Standard	70.9	67.6
P50	RT	MGI Standard	67.0	70.2
P51	RT	MGI Standard	66.8	71.6
**Average ± SD**			69.3 ± 12.2	70.2 ± 3.8

Additional File 2 Table 11. Number of processed reads and percentage of mapped reads by Salmon for muscle samples (N=51) sequenced at MGI, Latvia using standard reagents used in downstream DGE/GSEA analyses. Salmon indexing against the publicly available hg38 full decoy-aware salmon index. RP: Returning Participant; _V1 or _V2: RP Visit 1 or 2; C: Control; RT: Resistance Trained; RT-AS≤2: RT Exposed to AAS ≤2 weeks before sampling; RT-AS>10: RT Exposed to AAS >10 weeks before sampling.

Sample	Group	Sequencing Location & Chemistry	Processed Reads (Million)	Mapped Reads %
RP1_V1	RT-AS>10	MGI CoolMPS	50.6	75.7
RP2_V1	RT-AS≤2	MGI CoolMPS	64.6	72.7
RP2_V2	RP Visit 2	MGI CoolMPS	86.5	70.0
RP3_V1	RT-AS≤2	MGI CoolMPS	55.1	68.0
RP3_V2	RP Visit 2	MGI CoolMPS	71.0	68.3
RP4_V1	RT-AS≤2	MGI CoolMPS	79.0	71.2
RP4_V2	RP Visit 2	MGI CoolMPS	67.8	60.5
RP5_V1	RT-AS≤2	MGI CoolMPS	73.3	70.2
P01	RT-AS≤2	MGI CoolMPS	83.8	70.2
P02	RT-AS≤2	MGI CoolMPS	69.2	71.5
P03	RT-AS≤2	MGI CoolMPS	64.1	70.6
P04	RT-AS≤2	MGI CoolMPS	80.2	71.7
P05	RT-AS≤2	MGI CoolMPS	75.9	71.3
P07	RT-AS≤2	MGI CoolMPS	59.7	69.3
P08	RT-AS≤2	MGI CoolMPS	66.2	72.0
P09	RT-AS≤2	MGI CoolMPS	71.8	70.4
P10	RT-AS≤2	MGI CoolMPS	54.8	70.8
P12	RT-AS≤2	MGI CoolMPS	56.2	69.6
P13	RT-AS≤2	MGI CoolMPS	113.7	68.6
P06	RT-AS>10	MGI CoolMPS	60.8	70.2
P11	RT-AS>10	MGI CoolMPS	70.8	68.9
P14	RT-AS>10	MGI CoolMPS	74.8	67.8
P15	RT-AS>10	MGI CoolMPS	77.7	60.6
P16	RT-AS>10	MGI CoolMPS	78.6	71.0
P17	RT-AS>10	MGI CoolMPS	66.1	72.5
P18	RT-AS>10	MGI CoolMPS	71.5	71.7
P19	RT-AS>10	MGI CoolMPS	57.9	70.6
P20	RT-AS>10	MGI CoolMPS	71.8	67.9
P21	RT-AS>10	MGI CoolMPS	67.9	68.6
P23	C	MGI CoolMPS	70.6	65.1
P24	C	MGI CoolMPS	72.0	66.2
P26	C	MGI CoolMPS	68.2	71.1
P27	C	MGI CoolMPS	56.0	75.4
P28	C	MGI CoolMPS	40.6	67.5
P30	RT	MGI CoolMPS	57.1	80.5
P31	RT	MGI CoolMPS	55.4	70.3
P32	RT	MGI CoolMPS	56.2	69.7
P33	RT	MGI CoolMPS	65.6	72.7
P34	RT	MGI CoolMPS	73.0	69.4
P35	RT	MGI CoolMPS	56.4	75.8
P36	RT	MGI CoolMPS	71.1	73.2
P37	RT	MGI CoolMPS	91.4	66.6
P41	RT	MGI CoolMPS	74.6	66.7
P42	RT	MGI CoolMPS	60.8	71.1
P43	RT	MGI CoolMPS	73.8	73.0

P45	RT	MGI CoolMPS	71.1	79.9
P46	RT	MGI CoolMPS	70.1	67.4
P48	RT	MGI CoolMPS	59.3	77.5
P49	RT	MGI CoolMPS	67.8	68.8
P50	RT	MGI CoolMPS	67.0	70.2
P51	RT	MGI CoolMPS	61.0	72.9
**Average ± SD**			68.2 ± 11.6	70.5 ± 3.7

Additional File 2 Table 12. Number of processed reads and percentage of mapped reads by Salmon for muscle samples (N=51) sequenced at MGI, Latvia using CoolMPS reagents used in downstream DGE/GSEA analyses. Salmon indexing against the publicly available hg38 full decoy-aware salmon index. RP: Returning Participant; _V1 or _V2: RP Visit 1 or 2; C: Control; RT: Resistance Trained; RT-AS≤2: RT Exposed to AAS ≤2 weeks before sampling; RT-AS>10: RT Exposed to AAS >10 weeks before sampling.

Additional File 2 Table 13a. The number of genes identified for DGE from whole blood samples sequenced with standard chemistry reagents at UoB.

Tissue	Comparison	
Blood	RP2-5:<br>Visit 2 (n=4), Visit 1 (n=4)	Group:<br>C=7, RT=20, RT-AS≤2=14, RT-AS≥10=10
Low-expressed genes	14,636	13,637
Genes available for DGE analysis	14,353	15,352

Additional File 2 Table 13b. The number of genes identified for DGE from whole blood samples sequenced with standard chemistry reagents at MGI.

Tissue	Comparison	
Blood	RP2-4:<br>Visit 2 (n=3), Visit 1 (n=3)	Group:<br>C=6, RT=17, RT-AS≤2=12, RT-AS≥10=8
Low-expressed genes	14,314	13,374
Genes available for DGE analysis	14,675	15,615

Additional File 2 Table 13c. The number of genes identified for DGE from muscle samples sequenced with standard reagents at MGI.

Tissue	Comparison	
Muscle	RP2-4:<br>Visit 2 (n=3), Visit 1 (n=3)	Group:<br>C=5, RT=17, RT-AS≤2=15, RT-AS≥10=11
Low-expressed genes	13,639	12,302
Genes available for DGE analysis	15,350	16,687

Additional File 2 Table 13d. The number of genes identified for DGE from muscle samples sequenced with CoolMPS reagents at MGI.

Tissue	Comparison	
Muscle	RP2-4:<br>Visit 2 (n=3), Visit 1 (n=3)	Group:<br>C=5, RT=17, RT-AS≤2=15, RT-AS≥10=11
Low-expressed genes	13,645	12,332
Genes available for DGE analysis	15,344	16,657

DGE: differential gene expression; UoB: University of Brighton; RP: Returning Participant; RT-AS≤2: Resistance Trained participant who self-declared AAS exposure ceased ≤2 weeks before sampling; RT-AS≥10: Resistance Trained participant who self-declared AAS exposure ceased ≥10 weeks before sampling.

Additional File 2 Table 14a. Number of differentially expressed genes across different group comparisons for blood samples sequenced with standard chemistry reagents at UoB subjected to DGE analysis.

Comparison							
RP2-5 Visit 2 (n=4) *vs* Visit 1 (n=4)	RT (n=20) *vs* C (n=7)	RT-AS≤2 (n=14) *vs* C (n=7)	RT-AS≥10 (n=10) *vs* C (n=7)	RT-AS≤2 (n=14) *vs* RT (n=20)	RT-AS≥10 (n=10) *vs* RT (n=20)	RT-AS≤2 (n=14) *vs* RT-AS≥10 (n=10)	Up/Down regulation
0	0	0	0	0	1	0	Up
0	1	0	0	0	0	1	Down

Additional File 2 Table 14b. Number of differentially expressed genes across different group comparisons for blood samples sequenced with standard chemistry reagents at MGI subjected to DGE analysis.

Comparison							
RP2-4 Visit 2 (n=3) *vs* Visit 1 (n=3)	RT (n=17) *vs* C (n=6)	RT-AS≤2 (n=12) *vs* C (n=6)	RT-AS≥10 (n=8) *vs* C (n=6)	RT-AS≤2 (n=12) *vs* RT (n=17)	RT-AS≥10 (n=8) *vs* RT (n=17)	RT-AS≤2 (n=12) *vs* RT-AS≥10 (n=8)	Up/Down regulation
1	0	0	0	0	1	0	Up
10	2	1	0	0	0	0	Down

Additional File 2 Table 14c. Number of differentially expressed genes across different group comparisons for muscle samples sequenced with standard reagents at MGI subjected to DGE analysis.

**Comparison**							
RP2-4 Visit 2 (n=3) *vs* Visit 1 (n=3)	RT (n=17) *vs* C (n=5)	RT-AS≤2 (n=15) *vs* C (n=5)	RT-AS≥10 (n=11) *vs* C (n=5)	RT-AS≤2 (n=15) *vs* RT (n=17)	RT-AS≥10 (n=11) *vs* RT (n=17)	RT-AS≤2 (n=15) *vs* RT-AS≥10 (n=11)	Up/Down regulation
2	2	34	2	120	0	12	Up
0	3	28	0	52	0	7	Down

Additional File 2 Table 14d. Number of differentially expressed genes across different group comparisons for muscle samples sequenced with CoolMPS reagents at MGI subjected to DGE analysis.

**Comparison**							
RP2-4 Visit 2 (n=3) *vs* Visit 1 (n=3)	RT (n=17) *vs* C (n=5)	RT-AS≤2 (n=15) *vs* C (n=5)	RT-AS≥10 (n=11) *vs* C (n=5)	RT-AS≤2 (n=15) *vs* RT (n=17)	RT-AS≥10 (n=11) *vs* RT (n=17)	RT-AS≤2 (n=15) *vs* RT-AS≥10 (n=11)	Up/Down regulation
4	2	38	2	94	0	21	Up
0	2	41	0	50	0	17	Down

Genes with a false discovery rate (FDR) < 0.05 and fold change of 1.2 were reported as differentially expressed. DGE: differential gene expression; UoB: University of Brighton; RP: Returning Participant; C: non-resistance trained control group; RT: Resistance Trained control group RT-AS≤2: Resistance Trained participant who self-declared AAS exposure ceased ≤2 weeks before sampling; RT-AS≥10: Resistance Trained participant who self-declared AAS exposure ceased ≥10 weeks before sampling.

Additional File 2 Table 15a. Number of differentially expressed gene sets or pathways across different group comparisons for muscle samples sequenced with standard reagents at MGI subjected to GSEA analysis.

Comparison							
RP2-4 Visit 2 (n=3) *vs* Visit 1 (n=3)	RT (n=17) *vs* C (n=5)	RT-AS≤2 (n=15) *vs* C (n=5)	RT-AS≥10 (n=11) *vs* C (n=5)	RT-AS≤2 (n=15) *vs* RT (n=17)	RT-AS≥10 (n=11) *vs* RT (n=17)	RT-AS≤2 (n=15) *vs* RT-AS≥10 (n=11)	Gene Set or Pathway
0	0	0	0	0	0	0	Hallmark
0	0	1	0	15	2	0	KEGG
0	0	4	0	119	0	3	Reactome
1	2	49	0	439	0	17	GO BP
0	1	18	0	220	0	23	GO MF

Additional File 2 Table 15b. Number of differentially expressed gene sets or pathways across different group comparisons for muscle samples sequenced with CoolMPS reagents at MGI subjected to GSEA analysis.

Comparison							
RP2-4 Visit 2 (n=3) *vs* Visit 1 (n=3)	RT (n=17) *vs* C (n=5)	RT-AS≤2 (n=15) *vs* C (n=5)	RT-AS≥10 (n=11) *vs* C (n=5)	RT-AS≤2 (n=15) *vs* RT (n=17)	RT-AS≥10 (n=11) *vs* RT (n=17)	RT-AS≤2 (n=15) *vs* RT-AS≥10 (n=11)	Gene Set or Pathway
0	0	0	0	0	3	0	Hallmark
0	0	2	0	14	4	0	KEGG
2	0	2	0	89	0	1	Reactome
0	0	18	0	224	0	4	GO BP
0	0	9	1	136	0	8	GO MF

Gene sets or pathways with a false discovery rate (FDR) < 0.05 were reported as differentially expressed. GSEA: Gene Set Enrichment Analysis; UoB: University of Brighton; RP: Returning Participant; C: non-resistance trained control group; RT: Resistance Trained control group; RT-AS≤2: Resistance Trained participant who self-declared AAS exposure ceased ≤2 weeks before sampling; RT-AS≥10: Resistance Trained participant who self-declared AAS exposure ceased ≥10 weeks before sampling; GO BP: subset of GO biological processes; GO MF: subset of GO molecular functions.
